# Noggin4 is a long-range inhibitor of Wnt8 signalling that regulates head development in *Xenopus laevis*

**DOI:** 10.1038/srep23049

**Published:** 2016-03-14

**Authors:** Fedor M. Eroshkin, Alexey M. Nesterenko, Alexander V. Borodulin, Natalia Yu. Martynova, Galina V. Ermakova, Fatima K. Gyoeva, Eugeny E. Orlov, Alexey A. Belogurov, Konstantin A. Lukyanov, Andrey V. Bayramov, Andrey G. Zaraisky

**Affiliations:** 1 Shemyakin-Ovchinnikov Institute of Bioorganic Chemistry, Russian Academy of Sciences, Moscow, 117997, Russia; 2 Belozersky Institute of Physico-Chemical Biology, Lomonosov Moscow State University, Leninskie gory, 1/40, 119991 Moscow, Russia; 3 Institute of Protein Research, Russian Academy of Sciences, Pushchino, 142290 Moscow Region, Russia

## Abstract

Noggin4 is a Noggin family secreted protein whose molecular and physiological functions remain unknown. In this study, we demonstrate that in contrast to other Noggins, *Xenopus laevis* Noggin4 cannot antagonise BMP signalling; instead, it specifically binds to Wnt8 and inhibits the Wnt/*β* -catenin pathway. Live imaging demonstrated that Noggin4 diffusivity in embryonic tissues significantly exceeded that of other Noggins. Using the Fluorescence Recovery After Photobleaching (FRAP) assay and mathematical modelling, we directly estimated the affinity of Noggin4 for Wnt8 in living embryos and determined that Noggin4 fine-tune the Wnt8 posterior-to-anterior gradient. Our results suggest a role for Noggin4 as a unique, freely diffusing, long-range inhibitor of canonical Wnt signalling, thus explaining its ability to promote head development.

The secreted protein Noggin1 is a well-known embryonic inducer of the body axis, which is expressed in the Spemann organizer and has a primary function as a BMP antagonist^1^,^2^. All vertebrates, with the exception of mammals, have two Noggins in addition to Noggin1: Noggin2 and Noggin4^3^,^4^,^5^. It has recently been demonstrated that Noggin1 and Noggin2, which have approximately 60% similarity with each other, inhibit not only BMP but also Nodal/Activin and Wnt-beta-catenin signalling^6^,^7^.

These findings indicate that it is important to elucidate the functions of the third sub-family of Noggins, Noggin4, which comprises members with substantially less homology with other Noggins (approximately 35%) and thus may exhibit different molecular and physiological activity. In particular, Noggin4 has amino acid substitutions in evolutionarily conserved positions critical for BMP binding to Noggin1 (Fig. 1a)^1^. Injection of *Noggin4* mRNA into the ventral side of *Xenopus laevis* embryos does not induce secondary body axis formation^8^. Moreover, in contrast to *Noggin1 and 2*, which are expressed in very restricted body regions, *Noggin4* is expressed during gastrulation diffusely throughout the dorsal ectoderm and thereafter, beginning from neurulation, also in the axial mesoderm^4^ (Fig. 1b and Fig. S1a–g).

In this study, we confirmed the inability of Noggin4 to bind BMP4 and antagonise its signalling. We also demonstrated that in contrast to Noggin1 and Noggin2, Noggin4 does not interfere with the Nodal/Activin pathway. We also demonstrated that Noggin4 binds to Wnt8 and inhibits its signalling. This inhibitory activity of Noggin4 has been demonstrated to be essential for forebrain development. Using live imaging techniques based on the FRAP (Fluorescence Recovery After Photobleaching) assay and mathematical modelling, we measured the diffusion coefficients for each Noggin in the embryonic ectoderm and determined that Noggin4 freely diffuses in the intercellular space (IS) and functions as a unique long-range Wnt inhibitor. In addition, by using a specially developed mathematical approach, we directly estimated the constant of Noggin4 binding to Wnt8 in living embryos and confirmed that Noggin4 fine-tunes the Wnt8 signalling gradient in the anterior region of the embryo.

## Results

### Noggin4 cannot antagonise BMP signalling, but its activity is necessary for head development

In contrast to Noggin1, when Noggin4 is ectopically expressed on the ventral side of the *Xenopus laevis* embryo it is unable to manifest the Noggin classical effect, i.e., the induction of a secondary body axis (Fig. S1h,i’). This finding indicates the inability of Noggin4 to antagonise BMP signalling, the cessation of which is absolutely required for secondary axis formation^9^. The inability of Noggin4 to bind BMP4 was directly confirmed by co-immunoprecipitation (CoIP) under conditions in which both Noggin1 and Noggin2 bound BMP4 (Fig. S2a; see also Fig. 2G in^6^ for the binding of Noggin2 to BMP4). Additionally, in contrast to other Noggins, Noggin4 was unable to suppress phosphorylation of the intracellular effector of BMP signaling, Smad1 (Fig. S2b), and to inhibit the *in vivo* expression of the BMP-signalling reporter (Fig. S2c). Thus, these findings indicate that Noggin4 lacks the most well-known Noggin function, i.e., binding and antagonizing BMP.

**Figure 1:**
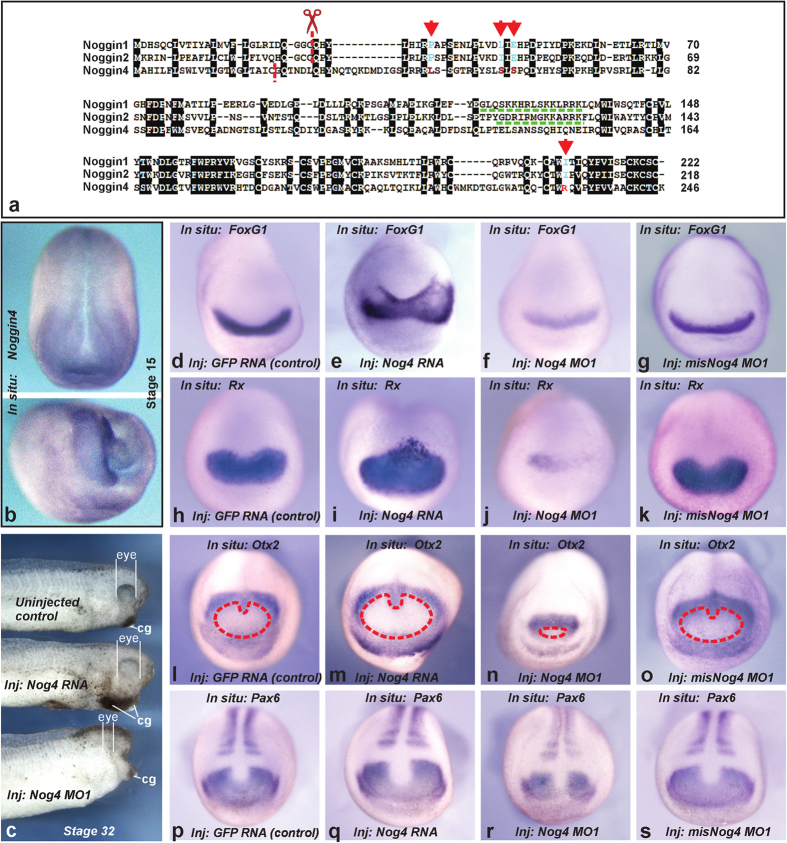
Properties of Noggin4. **(a)** Alignment of *Xenopus laevis* Noggins. Positions essential for Noggin1 binding to BMP^1^ are indicated by red arrows. HSPG binding motifs and cleavage sites are indicated by green and red dashed lines, respectively. **(b)** Whole-mount *in situ* hybridization of a midneurula embryo with dig-probe to *Noggin4* mRNA. Dorsal view at the top, the right-frontal view at the bottom. **(c)** Effects of *Noggin4* mRNA (80%, n = 120) and MO1 (85%, n = 130) injections on the development of head structures. Cg-cement gland. **(d–s)** *In situ* hybridization with dig-probes to *FoxG1*, *Rx*, *Otx2* and *Pax6* mRNA of midneurula embryos injected with *GFP* mRNA (control) (0%, n = 40; 0%, n = 50; 0%, n = 40; 0%, n = 30, abnormal phenotypes respectively), *Noggin4* mRNA (85%, n = 30; 61%, n = 35; 81%, n = 40; 65%, n = 40 abnormal phenotypes respectively), *Noggin4* MO1 (90%, n = 50; 86%, n = 40; 60%, n = 40; 60%, n = 40, abnormal phenotypes respectively) and *misNoggin4* MO1 (control) (0%, n = 30; 0%, n = 30; 0%, n = 40; 0%, n = 30, abnormal phenotypes respectively). Red dashed line surrounds the presumptive rostral forebrain territory. Anterior view, dorsal to the top.

We also identified enlargement of the head structures, including the eyes and the cement gland, in embryos that overexpressed Noggin4 (Fig. 1c). Converse effects were identified in embryos injected with *Noggin4* antisense morpholino oligonucleotide (MO) (Fig. 1c). Importantly, reduction in the anterior structures was demonstrated for both *MO1* and *MO2* variants of *Noggin4* morpholino, but not for the control, *misNoggin4 MO1* (Fig. S3a–c). The specificity of MO effects was confirmed by rescue experiments in which *Noggin4 MO1* was injected with *Noggin4* mRNA lacking the *MO1* target site (Fig. S4a).

Embryos injected with *Noggin4* mRNA demonstrated posterior and lateral expansion of the presumptive rostral forebrain territories marked by the expression of *FoxG1*, *Rx*, *Otx2* and *Pax6* (Fig. 1e,i,m,q). In contrast, the expression of these markers within the anterior neural plate was decreased in embryos injected with *MO1* or *MO2* (Fig. 1f,j,n,r; Fig. S3d–i).

Importantly, when *Noggin4* mRNA was injected into single blastomeres, the effects were identified also in regions other than the regions that contained the injected mRNA (Fig. S5), which indicates the non-autonomous activity of Noggin4.

### Noggin4 binds Wnt8 and antagonises its signalling

The ability of Noggin4 to promote the anterior phenotype, as well as its inability to antagonise BMP signalling suggests that Noggin4 may operate similarly to Wnt/*β*-Catenin and/or Activin/Nodal inhibitors, whose activity is essential for the head structure development^10^,^11^.

**Figure 2:**
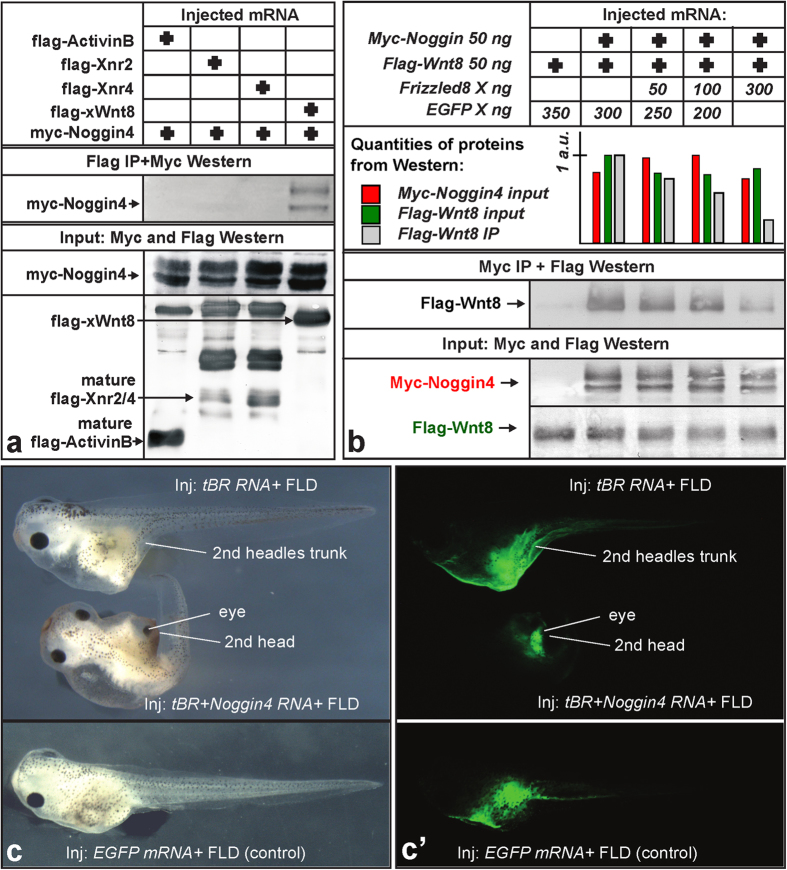
Noggin4 binds and antagonises Wnt8. **(a)** Analysis of Noggin4 interactions with ActivinB, Xnr2, Xnr4 and XWnt8 by CoIP assay. **(b)** Frizzled8 competes with Noggin4 for Wnt8 binding in the CoIP assay. Embryos were injected at the 2-cell stage with *Noggin4* and *Wnt8* mRNA or with these mRNAs mixed with increasing amounts of Frizzled8 mRNA as indicated. CoIP was conducted using crude extracts of the mid-gastrula embryos as described by^52^. See Methods section for the method of WB quantification. **(c)** Induction of a secondary head with a cyclopic eye by co-injection of *Noggin4* and *tBR* mRNA (60%, n = 70). *tBR* mRNA injected alone induces only a secondary trunk (80%, n = 70). No secondary axes were observed in the control embryos injected by *EGFP* mRNA (100%, n = 60). (c’) Location of co-injected Fluorescein-Lysin-Dextran (FLD) in tadpoles shown in (**c**).

Using CoIP and surface plasmon resonance (SPR) assays, we demonstrated that Noggin4 binds Wnt8, which is a principal ligand in Wnt/*β*-Catenin signalling that regulates the anterior-posterior patterning during gastrulation and neurulation (Fig. 2a; Fig. S6). The *K*_*d*_ of the Noggin4/Wnt8 complex was estimated via quantitative CoIP and SPR as 10 and 90 nM, respectively (see Materials and Methods and Fig. S6). In contrast, Noggin4 did not bind or suppress the activity of ActivinB or the Xenopus Nodal-related homologs Xnr2 and Xnr4 (Fig. 2a; Fig. S2d,e).

The inhibitory influence of Noggin4 on Wnt8 signalling was confirmed by its ability to down-regulate the specific *β*-catenin reporter TOPFlash, which is activated by Wnt8 (Fig. S2f). In contrast, Noggin4 did not antagonize the activity of *β*-catenin, the intracellular effector of canonical Wnt signalling, in a similar test (Fig. S2f). These findings indicate that Noggin4 may operate at the level of or upstream of Wnt8 receptors.

Indeed, using a CoIP assay, we confirmed that Noggin4 competed for Wnt8 with its receptor Frizzled8, which is expressed in the anterior neuroectoderm^12^ (Fig. 2b). The role of Noggin4 as a true endogenous inhibitor of Wnt signalling was demonstrated in experiments in which we observed activation of several direct genetic targets of the *β*-catenin pathway, including *Axin2*, *HoxA1*, *HoxB1*, *HoxD1*, *Siamois* and *Xnr3* 13, in embryos injected with *Noggin4* MO1 (Fig. S2g). Conversely, expression of these targets was inhibited in embryos injected with *Noggin4* mRNA (Fig. S2g).

Provided that Noggin4 directly inhibits Wnt/*β*-Catenin signalling, it may induce secondary heads in cooperation with a BMP inhibitor^14^. We identified secondary head structures, including forebrains with cyclopic eyes, in approximately 60% (n = 70) of the tadpoles co-injected with *Noggin4* mRNA and mRNA of the dominant-negative variant of the BMP receptor, tBR (Fig. 2c,c’). Together with the previously described findings, this result confirms the inhibitory influence of Noggin4 on Wnt/*β*-Catenin signalling.

### Noggin4 diffuses in living embryos at a substantially higher rate than other Noggins and Wnt8

To act as a natural antagonist of Wnt8, Noggin4 must spread and interact with Wnt8 within the IS in living embryos. To track Noggin4 and Wnt8 diffusion, we produced constructs that encoded secreted fusions of Noggin1/2/4 and Wnt8 with EGFP or TagRFP (Table S1; Fig. S7). Importantly, these fusion proteins retained abilities of their wild-type variants to influence BMP and Wnt signalling reporters (Fig. S8a,b) and to induce, with the collaboration of tBR, secondary heads with cyclopic eyes (Fig. S8c,d).

**Figure 3:**
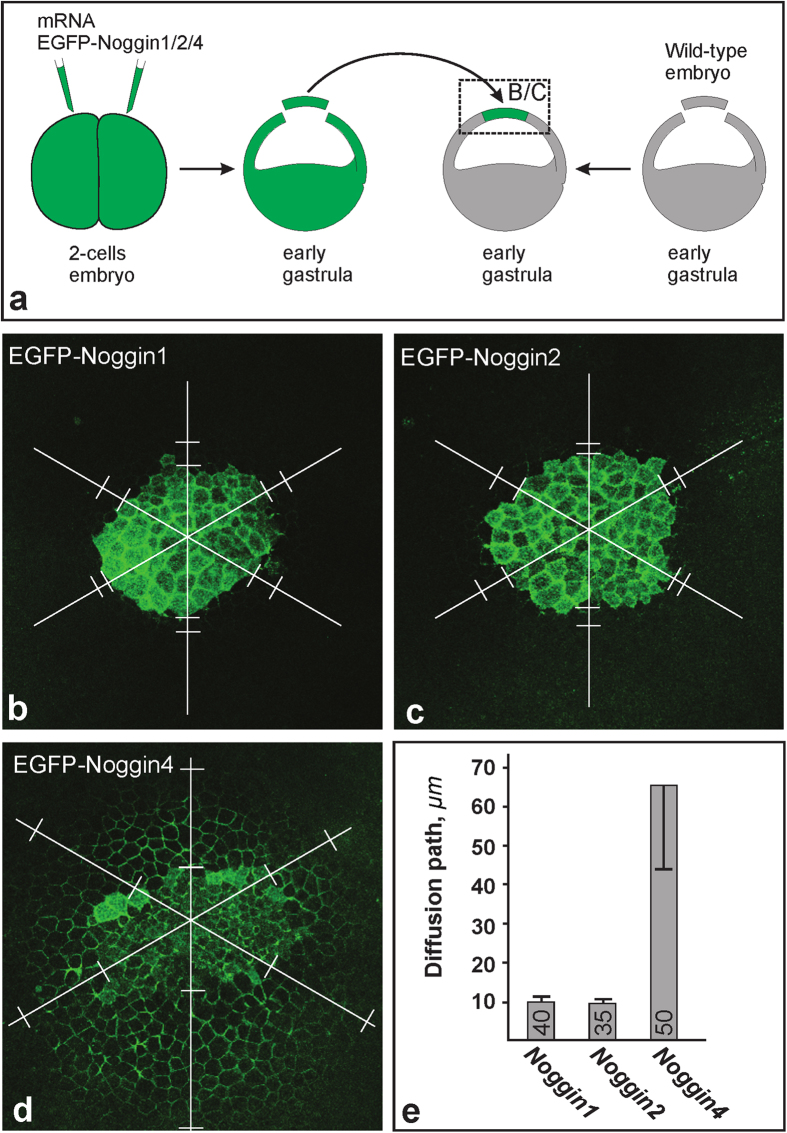
Diffusion of EGFP-Noggin1/2/4 from the ectodermal grafts. **(a)** Experimental design. The region that corresponds to images (**b**–**d**) is framed by a dashed rectangle. **(b–d)** Representative examples of the grafts 1 hour after transplantation. Note that in contrast to recipient embryo cells, graft cells contain EGFP-Noggin1/2/4 in the cytoplasm. To compare the diffusibility of the EGFP-Noggin1/24, the diffusion path (DP) was measured for each graft in six directions (white lines radiated under 60 degrees from the approximate centre of the graft), beginning from the graft border until the point at which the EGFP fluorescence level in an IS became equal to the background fluorescence level in the donor IS (pairs of white bars perpendicular to the radial lies). **(e)** Medium DP and deviation calculated for all grafts per type (number of grafts is indicated within each column).

First, we tested the diffusibility of the fusion proteins in a transplantation assay. We grafted small pieces of animal ectoderm that expressed EGFP-Noggin1/2/4 to the animal ectoderm of wild-type early gastrula embryos and measured the diffusion paths of secreted EGFP-Noggin1/2/4 in the recipient’s ectoderm one hour after grafting, as shown in Fig. 3a. The findings indicated that EGFP-Noggin4 diffused substantially faster than EGFP-Noggin1 and EGFP-Noggin2 (Fig. 3b–e).

**Figure 4:**
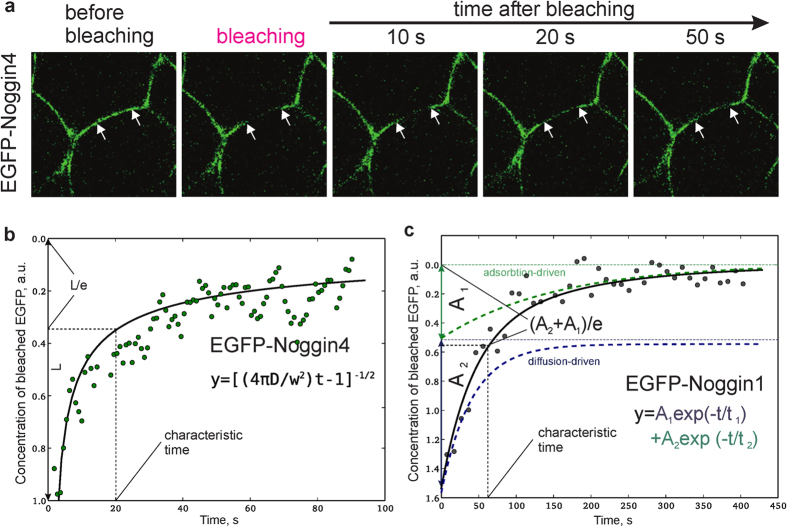
Analysis of Noggins and Wnt8 diffusivity. **(a)** Representative snapshot images of the photobleaching procedure of EGFP-Noggin4 in IS. Arrows indicate the margins of the bleached region. Snapshots for other studied proteins are presented at Fig. S9. Representative examples of EGFP-Noggin4 **(b)** and EGFP-Noggin1 **(c)** FRAP kinetics in single zones of the ectodermal IS. Equations and curves that fit the obtained data sets are shown under the curves.

Unfortunately, this assay allows only a rough comparison of the diffusivities because of the substantial variations in wound healing and the different sizes of the transplants. Therefore, we developed an approach based on the FRAP method, which enabled the measurement of the diffusion coefficient of a fluorescently labelled protein in a “single” IS between two adjacent cells in the embryonic ectoderm. Accordingly, the fluorescence of the proteins was bleached in a small linear zone (typically 7.5 *μ*m in length) of the IS using the laser beam of a confocal microscope. Immediately after bleaching, the FRAP was recorded, which reflected the lateral diffusion of the unbleached protein from outside the bleached zone (Fig. 4, Fig. S9 and Movie S1).

The FRAP data obtained for EGFP-Noggin4 fit well with the theoretical curve (Fig. 4b; Fig. S10) built by equation (1), which describes free diffusion in a one-dimensional space:


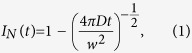


where *D* indicates the “true” Fickian diffusion coefficient, *I*_*N*_ indicates the normalized intensity of a fluorescent signal, and *w* indicates the linear size of the bleached zone (see Supplementary Materials and Methods, 1.1).

The value of *D*, which was calculated from this curve by measuring the FRAP characteristic time, was 3.8 ± 1.3 *μm*^2^/*s*; this value was consistent with the theoretical estimation of *D* for Noggin4 (8.0 *μm*^2^/*s*), which was determined by hydrodynamic modelling (Table 1; Supplementary Materials and Methods, 2; Fig. S11). Such a good agreement by an order of magnitude between Noggin4 diffusivity determined in a living embryo and theoretically calculated in an assumption of Noggin4 free diffusion is consistent with the lack of potential heparin binding motifs in Noggin4 (Fig. 1a). Otherwise, the diffusion in living embryos should be substantially slower because of interactions with heparan sulphate proteoglycans (HSPGs) in IS.

However, we discovered that equation (1) is not applicable to the FRAP kinetics of Noggin1, Noggin2 or Wnt8 (Fig. S10 and Supplementary Materials and Methods, 1.1 for details) potentially because heparin-binding motifs are present in these proteins, which result in their strong adsorption on HSPG^15^,^16^,^17^,^18^. We demonstrated that the FRAP kinetics in this case should be described by the sum of two exponents with two characteristic times: the diffusion-driven recovery time, *τ*_1_, and the adsorption-driven recovery time, *τ*_2_ (Fig. 4c and Supplementary Materials and Methods, 1.2 for details):


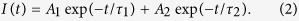


*A*_1,2_ indicate contribution coefficients of the diffusion- and adsorption-driven recovery in FRAP kinetics, respectively.

**Figure 5:**
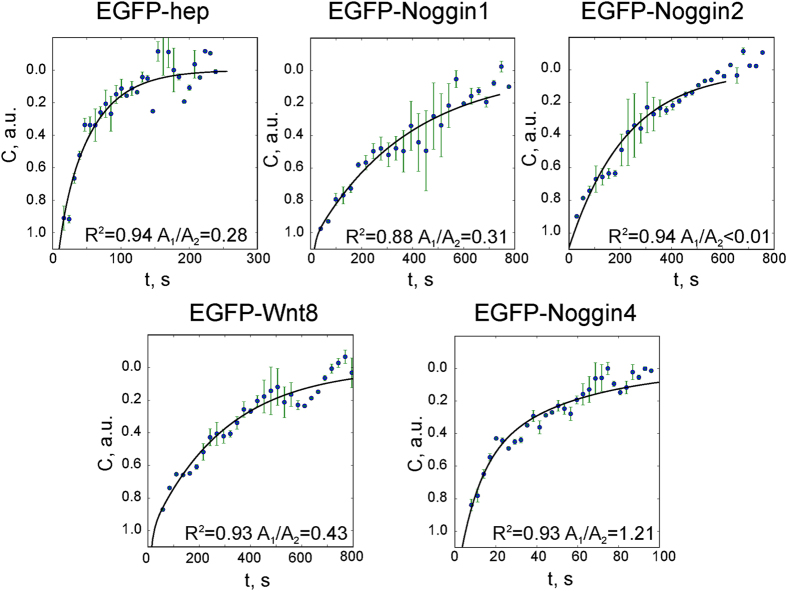
FRAP data fitted with a two-exponential equation. Averaged normalised data with error bars and approximation curves are plotted for each experiment. The determination coefficient (*R*^2^) is presented for each group of observations, as well as the weight of the fast exponent, *α* = *A*_1_/*A*_2_ (as shown in Fig. 4); this value reflects the ratio of free-to-adsorbed protein (analysis details are described in Supplementary Materials and Methods, 1.3).

The FRAP data for all examined proteins fitted by curves built with equation (2) are shown in Fig. 5.

Our diffusion modelling was hindered by adsorption and also demonstrated that the diffusion coefficient *D*, which is conventionally defined as the molar flux per unit of the concentration gradient, has no constant value and continuously changes in time and space. Therefore, to compare the diffusivities of proteins that move freely and with adsorption, we measured the fluorescence-recovery characteristic time for the diffusion processes hindered by adsorption; this characteristic time was considered to correspond to a virtual process of free diffusion (Supplementary Materials and Methods, 1.3). Accordingly, the diffusion coefficient of this virtual free diffusion, i.e., the effective diffusion coefficient, *D*_*E*_, was calculated by equation (5) (see Materials and Methods). The *D*_*E*_ values of Noggin1, Noggin2 and Wnt8 calculated with this approach were approximately 50-fold lower than the value for Noggin4 (Table 1).

Importantly, hydrodynamic modelling indicated that the fusion with EGFP did not significantly change the diffusion coefficients of Noggin1–4 compared with the untagged proteins (Table 1, Fig. S11 and Supplementary Materials and Methods, 2). This finding indicates that the diffusion coefficients calculated by the FRAP-based approach in living embryos provided good estimates of the *D*_*E*_ of endogenous Noggins.

### Visualisation of Noggin4 binding to Wnt8 in living embryos

**Figure 6:**
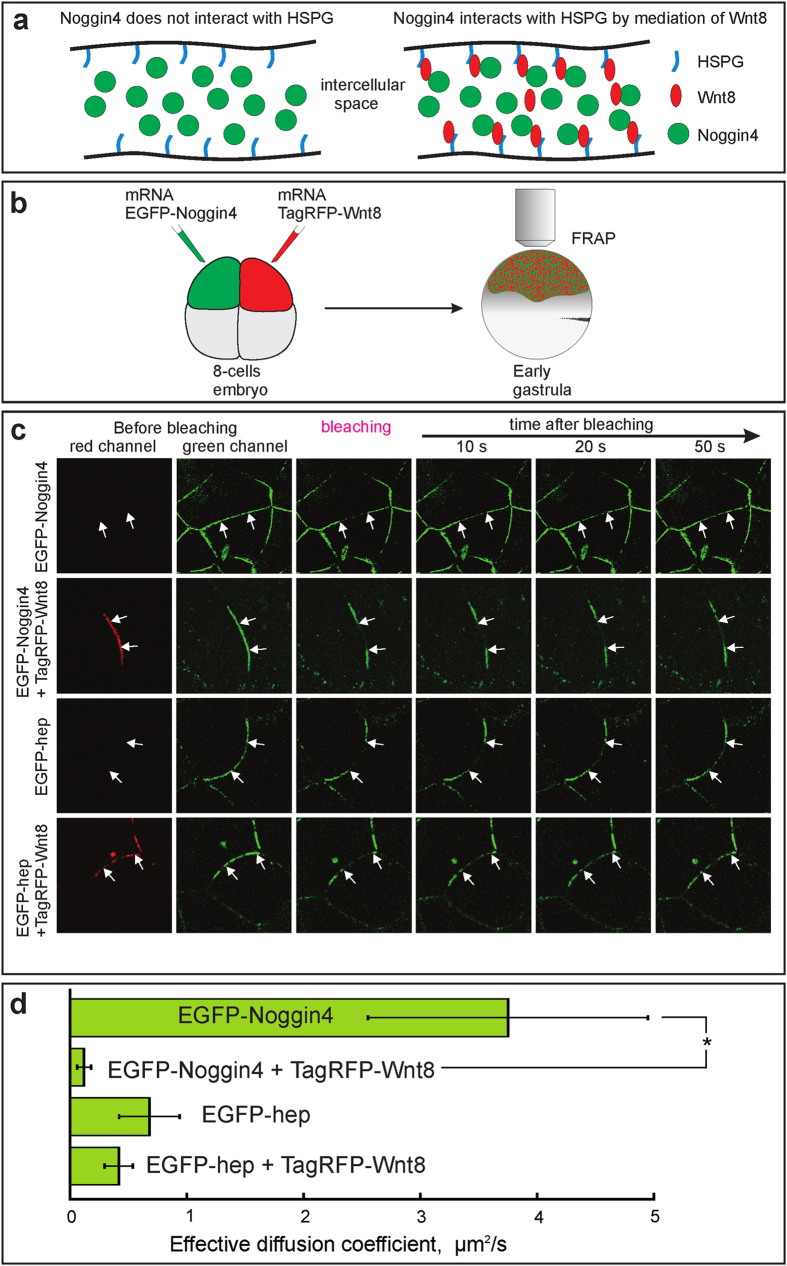
Investigation of Noggin4 interaction with Wnt8 in living embryos via FRAP assay. **(a)** The cartoon explains how free diffusion of Noggin4 is retarded by its interaction with Wnt8 adsorbed on HSPG. **(b)** Schema for obtaining embryos that express EGFP-Noggin4 and TagRFP-Wnt8 in different cells. **(c)** Representative examples of FRAP in the intracellular space of animal ectoderm in embryos injected, as shown in B, with mRNAs of indicated fluorescently labelled proteins. Arrows mark the borders of the bleached region. **(d)** Mean values of the effective diffusion coefficients of EGFP-Noggin4 and EGFP-hep measured experimentally and shown in (**a–c)**. Five to 7 series of experiments (10 to 20 individual measurements per experiment) were conducted for each of the four types of injections. Averaged FRAP curves are presented at Fig. S15.

We used the significant difference between the Noggin4 and Wnt8 diffusivities to directly test their interactions in living embryos by comparing the *D*_*E*_ of Noggin4 expressed alone or in the presence of Wnt8 (Fig. 6a). To this end, 70 pg of *EGFP-Noggin4* and *TagRFP-XWnt8* mRNAs were injected into adjacent blastomeres of 8-cell embryos, and the FRAP characteristic time was measured for the EGFP-Noggin4 in an IS that contained both proteins (Fig. 6b,c). Under these conditions, the *D*_*E*_ of EGFP-Noggin4 was approximately 30-fold lower than when *EGFP-Noggin4* mRNA was injected alone (Fig. 6d and Movie S2). Importantly, no significant decrease in the diffusion rate was identified when the secreted version of EGFP fused with the heparin binding site of Noggin1 (EGFP-hep) was subjected to FRAP alone or in the presence of TagRFP-XWnt8 (Fig. 6c,d). We used the fusion EGFP-hep because the secreted “pure” EGFP rapidly leaved the IS and could not be detected in our experimental conditions.

To further confirm that Noggin4 interacts with Wnt8 in living embryos, we used a transplantation assay (Fig. S12a indicates the experimental scheme). One hour after wound healing, the diffusion of EGFP-Noggin4 from the graft into the IS of the recipient animal ectoderm that expressed TagRFP-Wnt8 was visualised with a confocal microscope. The results indicated that the diffusion of EGFP-Noggin4 was significantly retarded in the recipient embryo IS, which expressed TagRFP-Wnt8, compared with the recipients that expressed TagRFP-hep (Fig. S12b,c). These experiments also confirm the interaction of Noggin4 with Wnt8.

### Estimation of the affinity of Noggin4 to Wnt8 in IS of living embryos

The model (3) describes the diffusion of a protein interacting with immobile components of the intercellular matrix. However, the same model could be used to describe diffusion of a protein, which interacts with another protein tightly adsorbed on the intercellular matrix. In this case, the ratio of amplitudes (*A*_2_/*A*_1_) of the fast and slow components in the FRAP kinetics of the diffusing protein described by equation (2) will be determined by the binding affinity of the interacting proteins, *K*_*d*_, as well as by the initial absolute concentrations of both proteins in the IS (Fig. S13 and Supplementary Materials and Methods, 3). We used these considerations to assess directly in a living embryo the affinity of EGFP-Noggin4 to TagRFP-Wnt8, which due to its extremely low mobility in the IS can be considered as quasi-immobile.

To measure absolute concentrations of EGFP-Noggin4 and TagRFP-Wnt8 in the IS, we first calibrated the fluorescence intensity *in vitro*, using different concentrations of the recombinant EGFP and TagRFP under the same microscopy settings as in the FRAP experiments (Fig. S14). On the basis of this calibration, the average concentrations of TagRFP-Wnt8 and EGFP-Noggin4 in the IS of embryos injected with 70 pg/blastomere of mRNA of each of the proteins at 8-cells stage were determined to be approximately 1 *μ*M. These values were substituted into equation (S1.5) (Supplementary Materials and Methods), and a *K*_*d*_ was selected that fit the experimental FRAP data (Fig. S15a). As a result, the *K*_*d*_ of the EGFP-Noggin4/TagRFP-Wnt8 protein complex was estimated to not exceed 100 nM, which is in agreement with estimations of this *K*_*d*_ obtained by qCoIP and SPR methods (see above).

### Noggin4 is able to fine-tunes the posterior-to-anterior Wnt8 signalling gradient in embryos

The posterior-to-anterior Wnt8 concentration gradient is critical for the patterning of the anterior neurectoderm in vertebrates^19^,^20^. In the *Xenopus* neural plate, *Wnt8* is expressed beginning from its posterior end and up to the presumptive hind-midbrain border, which corresponds to the posterior limit of the *Otx2* expression domain in the anterior neural plate^21^,^22^. Diffusing from this border, Wnt8 forms a gradient with a minimum at the anterior margin of the neural plate. To fine-tune this gradient in *Xenopus laevis* embryos, endogenous Noggin4 must compete with Frizzled receptor proteins for the binding to Wnt8.

**Figure 7:**
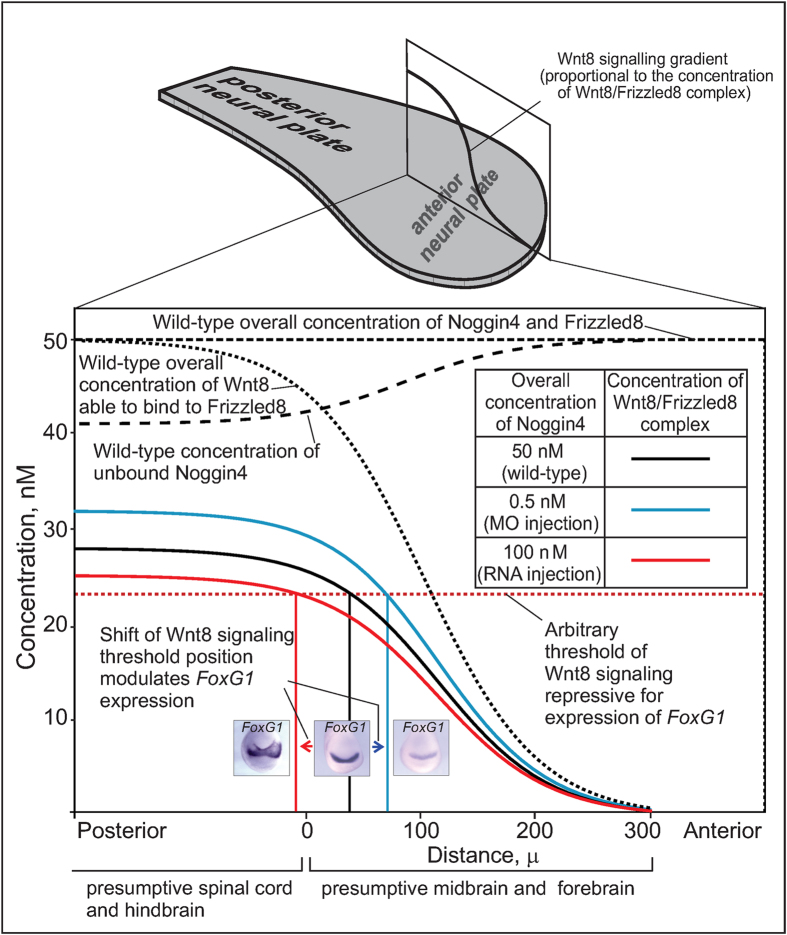
Mathematical modelling of the Noggin4 influence on the anterior-posterior Wnt8 signalling gradient. During neurulation, Wnt8 diffusing from the posterior regions within the anterior neural plate and interacting there with Frizzled8 forms a gradient of Wnt8 signalling, which is schematically presented by a solid black line within the rectangle standing along the neural plate midline on the schema shown above. Coloured curves within an enlarged version of this rectangle (below) demonstrate the theoretically calculated spatial distribution of the Wnt8/Frizzled8 complex (i.e., Frizzled8 receptor activated by Wnt8 binding) at three concentrations of Noggin4 (wild-type, decreased by Noggin4 MO injections and incresased by Noggin4 mRNA injections) indicated in the inserted table. Three inserted photos illustrate the assumed effects of the corresponding spatial shifts of the Wnt8/Frizzled8 complex arbitrary threshold concentration on the forebrain marker *FoxG1* expression. See main text, Supplementary Materials and Methods 4.2 and Fig. 1 for details.

We used mathematical modelling to analyse the consequences of the endogenous Noggin4 competition for Wnt8 binding with one of the main receptor of Wnt8 in the anterior ectoderm, Frizzled8^12^. First, we assessed the concentration of the endogenous Noggin4 protein in an embryo IS as approximately 50 nM (Supplementary Materials and Methods, 4.1, Fig. S16). Second, we supposed for simplicity that (i) Noggin4 is evenly distributed throughout the anterior neuroectoderm; (ii) Frizzled8 is also present at a total concentration of 50 nM throughout the anterior neuroectoderm; and (iii) the Wnt8 concentration forms a posterior-to-anterior gradient, which initiates from a maximum of 50 nM at the presumptive hind-midbrain border and then decreases to the anterior end according to a logistic sigmoid function (Fig. 7).

This sigmoid shape of the Wnt8 concentration gradient was postulated as the one that most resembled the shape of the experimentally measured gradient of Wnt/*β*-Catenin signalling in the presumptive neural plate^19^. Finally, we considered that the Noggin4 affinity for Wnt8 does not exceed 100 nM (as previously discussed), whereas the affinity of Wnt8 for Frizzled8 is 10 nM^23^. On the basis of these initial conditions, the effects of different concentrations of Noggin4 on the concentration gradient of the Wnt8-Frizzled complex along the midline of the *Otx2* expression area in anterior neural plate, the length of which was considered equal to 300 microns, were evaluated using equations (S4.1) and (S4.2) (Supplementary Materials and Methods, 4.2). This modelling mimicked the results of our experiments on the up- and down-regulation of Noggin4 by injecting its mRNA or MO. These virtual changes in the Noggin4 concentration spatially shifted an arbitrary threshold of the Wnt8 signalling gradient by a range of approximately 100 microns (Fig. 7). Thus, our experiments and modelling both confirm that Noggin4 plays a role as a long-range inhibitor of Wnt8 signalling that regulates the posterior-anterior Wnt signalling gradient in wild-type embryos.

## Discussion

We present several lines of evidence that Noggin4 operates as a long-range inhibitor of a principal ligand of the canonical Wnt signalling pathway, Wnt8. The qCoIP and SPR experiments demonstrated that Noggin4 most likely executes this function directly binding to Wnt8, which blocks its interaction with the Wnt receptor Frizzled. In this respect, Noggin4 is reminiscent of the Wnt8 antagonist Frzb/sFRP3; however, it is different from another Wnt inhibitor, Dkk, which does not directly bind to Wnt, but operates by binding to the Frizzled co-receptor Lrp5/6 24. Moreover, Noggin4 resembles Frsb/sFRP3 in its ability to induce, in collaboration with BMP inhibitors, a secondary head with a single eye, which was explained by the ability of Frzb to selectively inhibit Wnt8, but not Wnt3a^25^,^26^,^27^,^28^. In contrast, Dkk is an antagonist of both Wnt8 and Wnt3a and induces a secondary head with bilateral eyes in a similar test^10^,^26^. It would be interesting to verify whether Noggin4, like Frzb, is also unable to antagonise Wnt3a activity.

During this study, we developed an approach to investigate the diffusion of secreted proteins in the IS of living embryos. The diffusivity of fluorescently tagged morphogens in embryonic tissues is typically studied via the analysis of FRAP kinetics in a square area that contains many cells^29^,^30^. However, to correctly calculate the diffusion coefficient, the dimensionality of the diffusion space should be determined^31^. Meanwhile, the IS network usually has a dimensionality that is not an integer value; thus, this may not be a trivial task^32^. Moreover, the process of recording FRAP kinetics during such experiments typically requires substantial time: up to dozens of minutes. Thus, side processes, such as protein degradation and/or secretion, may begin to affect the fluorescence recovery and diffusion and should therefore be considered in a proper calculation of diffusivity^33^. Our approach lacks the aforementioned disadvantages because it enables direct assessment of the real diffusion rate via measurement of the FRAP half-life in a “single” IS, which significantly reduces the measurement time and simplifies further calculations.

We determined that the diffusivity of Noggin4 exceeds the diffusivities of Noggin1, Noggin2 and Wnt8 by more than an order of magnitude. Although no quantitative data on the diffusivities of other known protein Wnt inhibitors are available, it appears likely that these properties of Noggin4 make it unique among these inhibitors because all of them, including DAN, Dkk, Cerberus and Frzb/SFRPs, contain heparin binding motifs and thus, their diffusion in the IS should be retarded^34^,^35^,^36^,^37^.

Notably, the diffusivities determined in this study for Wnt8 and Noggin1/2/4 (from 0.05 to 3.8 *μ*^2^2/s) are of the same order of magnitude as those previously measured by FRAP and Fluorescence Correlation Spectroscopy (FCS) methods for other secreted proteins in *Drosophila*, *Danio* and *Xenopus* embryos (from 0.05 to 11 *μm*^2^/s^29^,^38^,^39^,^40^). This coincidence clearly confirms the validity of our results. *D* values an order of magnitude larger have been obtained in some studies using the FCS method. For example, a *D* of 58 *μm*^2^/s has been reported for Fgf8-EGFP measured in zebrafish embryos^41^. In our view, this discrepancy could be explained by the arbitrary nature of the interpretation of the autocorrelation function during FCS data processing. It is often presented as an autocorrelation function of a virtual model system that comprises slow and fast particles, whose diffusion is assumed to be free and independent. In the work^41^, the authors assert that the diffusivity of the fast fraction is a more reasonable estimate of Fgf8 diffusion. However, this estimate, which is close to the theoretical value for the diffusion of Noggin-size proteins in pure water (Fig. S11b), appears unrealistic because the actual viscosity of the intercellular fluid should be higher than the viscosity of water. Moreover, the diffusion of Fgf8 should be seriously retarded in an IS because of the high affinity of Fgfs for HSPG^42^.

According to three methods, qCoIP, SPR and the *in vivo* FRAP-based approach, Noggin4 binds to Wnt8 with moderate affinity characterised by a *K*_*d*_ between 10–100 *nM*. Obviously, this *K*_*d*_ value is higher than that reported for Wnt8 compex with Frizzled8 or Frzb/sFRP3 (10 nM)^23^,^28^. However, our mathematical modelling demonstrated that even the *K*_*d*_ value of 100 nM enables Noggin4 to effectively regulate the Wnt8 signalling gradient that governs the anterior-posterior patterning of the neural plate.

Importantly, in modelling the effect of Noggin4 on the Wnt8-signalling gradient, we have postulated the gradient itself as an initial condition. Therefore, predictions on the basis of the modelling are independent of a specific mechanism of gradient formation. Specifically, it could be generated by the recently proposed cytoneme-based mechanism^43^,^44^. According to this mechanism, Wnt8 located on the tips of thin cell filopodia, referred to as cytonemes, induces Wnt8 signalling via the induction of clustering of the Lrp6-signalosome on the contact surface of recipient cells. Given our findings regarding Noggin4 binding to Wnt8 and its interference with Wnt signalling, it would be interesting to address a potential role for Noggin4 in blocking signalosome formation and/or function.

## Methods

Animal experiments were performed in accordance with guidelines approved by the Shemyakin-Ovchinnikov Institute of Bioorganic Chemistry (Moscow, Russia) Animal Committee and handled in accordance with the Animals (Scientific Procedures) Act 1986 and Helsinki Declaration.

### DNA constructs, luciferase assay and qRT-PCR

Cloning strategies are described in Table S1. The luciferase assay was performed as described^6^. Briefly, embryos were injected at 2–4 cell stage with the indicated on corresponding figures amounts of synthetic mRNA mixed with one of the luciferase reporter plasmids: GL3-ARE-Luc^45^; TOPflash (Millipore); TCFm-Luc^46^ and the reference pCMV-*β*-GAL plasmid (50 pg/embryo of each reporter plasmid). Animal cap explants were excised from the injected embryos at stage 10, cultured till stage 11, selected in three replicate samples by 10 explants in each and processed for luciferase analysis according to Promega protocol. Similar triplicate samples of the dorsal marginal zone explants of embryos injected with *Noggin4* MO1 or *misNoggin4* MO1 (control) were subjected to qRT-PCR with primers to *Axin2*, *HoxA1*, *HoxB1*, *HoxD1* (see Table S1), *Siamois*, *Xnr3* and two housekeeping genes, *Ef-1alpha* and *ODC*^47^,^48^.

### Synthetic mRNA, morpholino oligonucleotides and *in situ* hybridisation

Synthetic mRNAs were prepared by mMessage Machine SP6 Kit (Ambion) after linearisation of pCS2-based plasmids with NotI or pSP64-based plasmids with AseI. Two variants of morpholino antisense oligonucleotides (GeneTools) to *Noggin4* (*Noggin4* MO1 and MO2) and the control, mismatched, variant of Noggin4 MO1 (see Table S1) were injected with the final concentration 0.4 mM and the drop volume of 3–4 nl. All mRNAs and MOs were mixed with Fluorescein Lysine Dextran (FLD) (Invitrogen, 40 kD, 5 *μ*g/*μ*l) before injections. Whole-mount *in situ* hybridisation was performed as described^49^.

### Immunoprecipitation and antibodies

To obtain secreted Flag- and Myc-tagged proteins for CoIP, the embryos, preliminary injected with synthetic mRNA encoding Flag- and Myc-tagged proteins, were released from envelopes at the early gastrula stage, dissociated in 10 *μ*l per embryo of CMFM (calcium/magnesium-free medium: 88 mM NaCl, 1 mM KCl, 2.4 mM NaHCO_3_, 7.5 mM Tris-HCl, pH 7.6, 0.1% BSA) and allowed to secrete proteins into the medium during 2 hr at room temperature on a rotating plate. Then, 2 mM EDTA and protease inhibitor cocktail (Sigma) were added, and incubation was continued for one hour until embryos completely dissociated to separate cells. These cells were gently sedimented (1000 rpm, 3 min), supernatant was supplemented with 0.9 mM CaCl_2_, 0.33 mM MgCl_2_, 0.1% NP40, 2 mM reduced glutathione and 2 mM oxidised glutathione (conditioned CMFM + IP additives) and used as input for immunoprecipitation. In the case of Myc-Noggin1, Myc-Noggin2 and Myc-Noggin4, 1.5 ml of conditioned medium was mixed with 150 *μ*l of EZview Red ANTI-MYC Affinity Gel (Sigma E6654) and incubated for 2 hr at 4 °C on a rotating wheel followed by 3 washes with CMFM + IP additives. Conditioned media, containing Flag-Activin, Flag-BMP4, Flag-Xnr3, Flag-Xnr4 or Flag-Wnt8, were preliminary concentrated 5 times at Amicone Ultra (Ultracel-10k) centrifugal Filter Units. Then, aliquots of the concentrated conditioned CMFM + IP additives, containing desired amounts of Flag-tagged protein, were mixed either with 50 *μ*l aliquots of EZview Red ANTI-MYC Affinity Gel equilibrated with 0.5% BSA (control) or with 50 *μ*l aliquots of EZview Red ANTI-MYC Affinity Gel containing bound Noggin1, Noggin2 or Noggin4 (see above) and incubated at 4 °C overnight on a rotating plate. After 5 washes with CMFM + IP additives, protein complexes were removed from ANTI-MYC Affinity Gel by incubation with 0.15 mg/ml c-MYC peptide (SigmaM2435) in CMFM + IP additives and analysed by Western blots as described previously^6^. Detection of P-Smad1, Smad1 and alpha-tubulin in embryos was performed by Western blotting with the corresponding antibodies to these proteins and according to the manufacturer’s protocols (see legend to Fig. S2b for more details). Amount of protein at the Western blot was measured with ImageJ software as integral density after channel splitting and background subtraction.

### Quantitative co-immunoprecipitation

To measure *K*_*d*_ of Wnt8-Noggin4 complex by quantitative co-immunoprecipitation (qCoIP), Flag-Wnt8 (5 *μ*g totally) and Myc-Noggin4 (7 *μ*g totally) were isolated as described in the previous section from two batches of embryos, 700 embryos in each, which were injected with the corresponding synthetic mRNA. For qCoIP assay, several gradually decreasing concentrations of Flag-Wnt8 were added to the standard amount of Myc-Noggin4 pre-mixed with anti-Myc resin (experimental samples) or to pure anti-Myc resin (control samples). Thereafter, concentrations of bound and unbound Flag-Wnt8 were measured in the quantitative Western Blotting (WB), by loading experimental and control samples on the same gel with serial dilutions of the standard sample–50 kDa amino-terminal Flag-BAP fusion protein (Sigma P-7582) (Fig. S6a,b). The blot scans were processed in ImageJ^50^ for quantitative measurements. Amounts of Wnt8 in the flow-through and in the eluate calculated from WB images were normalised to dilution and to the volume of the precipitation reaction to calculate the concentrations of unbound and bound fraction of Wnt8 (Table S2).

Theoretical analysis of Western blot data was performed as described in Supplementary Materials and Methods, 5.

### Analysis of Noggin4 binding to Wnt8 by Surface Plasmon Resonance (SPR) assay

To obtain Noggin4 protein in the amount sufficient for SPR assay, we purified it from the medium, to which it was secreted by HEK293 cells stably transformed by the plasmid expressing 3Myc-His6-Noggin4. To obtain this cell line, HEK293 cells were transfected with pCMV-3Myc-6His6-Noggin4 plasmid and grown in DMEM (Gibco BRL) supplemented with 10% heat-treated fetal calf serum (Hyclone), 100 units/mL Penicillin, 100 *μ*g/mL Streptomycin and 0.5 mg/mL Geneticin (Gibco BRL, Grand Island, NY) at 37 °C in 5% CO2 atmosphere. The selected clones were characterised by using immunoblotting with anti-His antibody. In order to obtain Noggin4, cells were plated and grown for two days; then the culture medium was collected and 3Myc-His6-Noggin4 was isolated from the medium using chromatography on Ni-Sepharose (GE Healthcare). After the buffer was changed to PBS by dialysis, the preparation was frozen in liquid nitrogen and kept at −70 °C until use. The *Xenopus laevis* recombinant Wnt8 labeled by 6-Hys-tag at the N-terminus and produced in yeast was obtained form MyBioSource, Inc., USA (Cat. N: MBS1208564).

The SPR measurements were performed on Biacore T200 apparatus. Ligand (3Myc-6His-Noggin4) was immobilised on CM5 chip (1,500 RU) using an amino-coupling kit according to the manufacturer’s instructions. All of the analyte binding measurements were performed with HBS-EP as the continuous running buffer at 25 °C. For the generation of concentration-dependent binding curves, 6His-Wnt8 was injected at various concentrations as indicated, and the response levels at equilibrium were plotted against the protein concentration. The equilibrium dissociation constants (*K*_*d*_) were obtained via a nonlinear least-squares fit of the binding curves to the Langmuir binding model describing a 1:1 binding stoichiometry, which finally resulted in the mean *K*_*d*_ = 90 ± 30 nM (Fig. S6C).

### Analysis of the protein diffusion by transplantation assay

Embryos were injected at 2–4-cell stage with a corresponding synthetic mRNA encoding a fluorescently labeled protein of interest (70 pg/blastomere) and at the beginning of gastrulation small pieces of their animal ectoderm were transplanted to the wild-type embryos of the same stage in 0.2 × MMR solution by using an eye micro-knife and a glass rod. From 10 to 20 transplantations were done for each of the analysed fluorescently labeled proteins in each series of experiments (in one day). From 3 to 6 series were performed in total for each of the proteins. After transplantation, the embryos were incubated for different time intervals at room temperature in 0.2 × MMR, and diffusion of the fluorescently labeled proteins was examined under the confocal microscope.

### Calibration of the confocal microscope

To assess the absolute concentrations of fluorescent proteins in IS, we performed calibration of the confocal microscope. To this end, stock solutions of pure recombinant EGFP or TagRFP (a gift from Evrogen) was diluted with 0.1 × MMR and put into the same 35 mm glass bottom culture dishes (MatTek) that were used for our FRAP experiments. Four different concentrations were probed for each protein. Note, that we obtained calibration curves with a wide spectrum of microscopy settings (under different pinhole, line average number, etc.). Pure 0.1 × MMR was set as a zero point. The relative signal density was measured in every point (summary signal divided to area). Representative examples of curves that were used for *in vivo* concentration analysis are shown on Fig. S14.

### Confocal microscopy and FRAP experiments

All confocal images and FRAP experiments were performed with the confocal microscope “Leica DM IRE 2” using HCX PL APO 63x objective, Ar/Kr laser (488 nm) for excitation of EGFP-tagged proteins, He/Ne laser (543 nm) for excitation of TagRFP-tagged proteins, and dichroic mirror DD488/543. The confocal images were obtained using the early-midgastrula stage (stages 10–11) embryos preliminary injected at 4–8 cell stage with synthetic RNA templates (70 pg/blastomere). From 20 to 30 rounds of FRAP recording were done for each type or combination of studied proteins. Each such round was done for the individual linear IS between two adjacent ectodermal cells on the animal hemisphere of the early gastrula. From 2 to 3 different ISs were analysed by this way in the same embryo.

As our preliminary FRAP experiments showed, a bleached region was often displaced from its initial position due to collective movement of the ectodermal cells. Therefore, a special Python script was developed to superimpose the successive images: http://erg.biophys.msu.ru/wordpress/archives/644.

### Mathematical modeling of diffusion

In the case of freely diffusing Noggin4, FRAP kinetics were fitted with the equation derived from the previously described equation for the one-dimensional diffusion^51^ (Supplementary Materials and Methods, 1.1). For modeling the diffusion with adsorption, we describe binding of the fluorescently labeled protein A with the immobile substance B, resulting in formation of the complex C, by the following kinetic scheme:


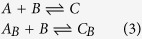


where subscript “B” means “bleached”. As we have shown, the fluorescence intensity decay can be described as a sum of two exponents with two characteristic times (S1.9) (Supplementary Materials and Methods). If we denote by *k*_−1_, *k*_1_ the dissociation and association rate constant, respectively, by *K* we denote the binding constant, by *σ*_*free*_ — the concentration of the free binding centers, by *w* — size of the bleach spot, then we have:


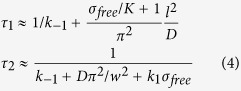


It is logically to call these two characteristic times as “adsorption-driven” and “diffusion-driven” characteristic times, respectively. Effective diffusion coefficients *D*_*E*_ were obtained according to the equation:


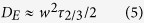


where *w* has the same meaning; *τ*_2*/*3_ is the time of fluorescence recovery to 2/3 of the equilibrium value (determined after fitting data with the two-exponential curve). The detailed derivation of the physical meaning of *D*_*E*_ and analysis of the corresponding reaction-diffusion system is provided in Supplementary Materials and Methods, 1.3.

### Modeling of Noggin4 effects on the Wnt8-signaling gradient

To describe interactions of Wnt8 with its receptor Frizzled8 and with Noggin4 the following reactions were considered:


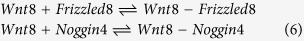


According to our estimation, the concentration of the endogenous Noggin4 in the ectodermal IS of the wild-type embryo at midgastrula stage is approximately 50 nM (Supplementary Materials and Methods, 4.1; Fig. S16). We also postulated, for simplicity, that (i) Frizzled8 has the same concentration of 50 nM; (ii) Frizzled8 and Noggin4 are distributed evenly along the anterior-posterior axis of the embryo; (iii) the Wnt8 concentration forms a posterior-to-anterior gradient, which starts from 50 nM at the posterior end of the embryo and declines according to a logistic sigmoid function up to zero at its anterior end (Supplementary Materials and Methods, 4.2). Using these considerations, *K*_*d*_ values of Noggin4/Wnt8 and Frizzled8/Wnt8 complexes, as well as our estimation of absolute concentration of each protein, we analysed numerically the effects of different concentrations of Noggin4 upon spatial distribution of Wnt8/Frizzled8 complex and, thus, upon Wnt8 signalling. Corresponding equations are presented in Supplementary Materials and Methods, 4.2.

## Additional Information

**How to cite this article**: Eroshkin, F. M. *et al.* Noggin4 is a long-range inhibitor of Wnt8 signalling that regulates head development in *Xenopus laevis*. *Sci. Rep.* **6**, 23049; doi: 10.1038/srep23049 (2016).

## Supplementary Material

Supplementary Information

Supplementary Movie S1

Supplementary Movie S2

## Figures and Tables

**Table 1:** Effective diffusion coefficients determined in FRAP experiments (*D*_*E*_) and the diffusion coefficients calculated by using hydrodynamic modeling (using viscosity of 0.05 Poises).

	EGFP-Noggin1	EGFP-Noggin2	EGFP-Noggin4	EGFP-Wnt8	EGFP-hep	EGFP-hep + Wnt8	EGFP-Noggin4 + Wnt8	Noggin1
FRAP data	0.05 ± 0.03	0.05 ± 0.03	3.75 ± 1.3	0.08 ± 0.03	0.68 ± 0.26	0.42 ± 0.12	0.12 ± 0.06	—
Hydro-dynamic	8.0	8.0	8.0	—	17.9	—	—	11.4

All values in *μm*^2^/s.
