# Supplementary material for: Noggin4 is a long-range inhibitor of Wnt8 signalling that regulates head development in Xenopus laevis
Source: Sci Rep. 2016 Mar 14;6:23049. doi: 10.1038/srep23049 (PMC4789793; doi:10.1038/srep23049)
Supplement: Supplementary Information [file srep23049-s1.pdf]

Supplementary information: Noggin4 is a long-range inhibitor of Wnt8 signalling that regulates head development in *Xenopus laevis*

Fedor Eroshkin[1], Alexey Nesterenko[1,2,*], Alexander Borodulin[1], Natalia Martynova[1], Galina Ermakova[1], Fatima Gioeva[1], Eugeny Orlov[1], Alexey Belogurov[1], Konstantin Lukyanov[1], Andrey Bayramov[1,*], and Andrey Zaraisky[1,*]

[1]Shemyakin-Ovchinnikov Institute of Bioorganic Chemistry, Russian Academy of Sciences, Moscow, 117997, Russia.
[2]Belozersky Institute of Physico-Chemical Biology, Lomonosov Moscow State University, Leninskie gory, 1/40, 119991 Moscow, Russia
[3]Institute of Protein Research, Russian Academy of Sciences, Pushchino, 142290 Moscow Region, Russia
[*]Correspondence to: azaraisky@yahoo.com; comconadin@gmail.com or andrbayr@gmail.com

**Name**	**Description and sequences of the morpholino oligonucleotides**
Noggin4 MO1	A common MO to positions -20–+5 of *Noggin4* mRNA of both *Xenopus laevis Noggin4* pseudoalleles:<br>5'–ACCATTATTCCTGTCTTGGAGATTA;
Noggin4 MO2	An equimolar mixture of two MOs to positions +1–+25 of Noggin4 mRNA of both *Xenopus laevis Noggin4* pseudoalleles:<br>5'-GATTATAGAAGTGAGTATCTTCCAT and<br>5'-ATGATATAACTAGTATGTGTACCAT.
misNoggin4 MO1	A mismatched variant of *Noggin4 MO1*:<br>5'-TCCAGTATACCTATCTAGGATATTC as a negative controls.
	**PCR primers and cloning strategy used to prepare DNA templates for generation of synthetic mRNA**
**Primers for qRT-PCR**	NP_001087841.1 *Xenopus laevis Axin2* mRNA<br>Forward: CCGCCGCTGTCGCATAA;<br>Reverse: CAGGTGGACGCAAAGAAGT.<br>Product length: 124 bp<br><br>NM_001085719.1 *Xenopus laevis homeobox A1 (hoxa1a)*, mRNA<br>Forward: CCATTTCCCCATCAGCCAGT;<br>Reverse: ATGTCCCCAGCCTTCTAACC.<br>Product length: 142 bp<br><br>FJ422584.1 *Xenopus laevis homeobox protein Hoxb-1 (Hoxb1)* mRNA<br>Forward: TCCAACACTGGACCCAACTC;<br>Reverse: CTTGGTGCTCCTGCCCATAA.<br>Product length: 97 bp<br><br>NM_001090566.1 *Xenopus laevis homeobox D1 (hoxd1)*, mRNA<br>Forward: TTTATGCCAGTCGGGGGTGT;<br>Reverse: ATCGTAATCGGGGCTGCTGT.<br>Product length: 150 bp
ActivinB,<br>*FlagActivinB,*<br>*FlagBMP4,*<br>*FlagXnr4,*<br>*MycNoggin1,*<br>*MycNoggin2,*<br>*Noggin1,*<br>*Noggin2,*<br>*Noggin4,*<br>*tBR,*<br>*Xnr2,*<br>*Xnr4,*<br>*XWnt8,*<br>*XWnt8-Flag*	These plasmids are described in[1] and.[2]
pCSβCatenin	This plasmid was kindly provided by Robert Grainger.

pCSFrizzled8	cDNA encoding full reading frame of *Frizzled8* was obtained by RT-PCR from the *Xenopus laevis* gastrula RNA with the following primers (*Frizzled8* coding sequences are underlined):<br>Frizzled8-EcoRI-forward<br>ATTGAATTCCACCATGGAGAGTCTGTCGCTGTCG<br>Frizzled8-XhoI-reverse<br>AATCTCGAGCATCTAGACCTGAGATAAGGGCA<br>Obtained cDNA was cloned into pCS2+ plasmid by EcoRI and XhoI. The activity of mRNA synthesised on this template was confirmed by its ability to induce secondary axes.
5'-UTR-MycNoggin4	**1st step.** Generation of 5'-fragment of MycNoggin4 cDNA containing the target site for Noggin4 MO at 5'-UTR. PCR from pNoggin4[1] with forward primer<br>5'-AATGGATCCTAATCTCCAAGACAGGAATA ("Noggin4 5'-UTR")<br>and a mixture of two reverse primers taken in ratio of 1:10 pM, respectively (Myc-tag-encoding sequences are underlined):<br>5'-CAGATCCTCTTCAGAGATGAGTTTCTGCTCATTTGTTTGGCCACAGATAGC ("Myc-Noggin4 reverse") and<br>5'-GAGGTCTTCCTCCGATATCAGCTTCTGTTCCAGATCCTCTTCAGAGATG ("Myc reverse").<br>**2nd step.** Obtaining of 3'-fragment of MycNoggin4 cDNA. PCR from pNoggin4 with a reverse primer 5'-TATCTCGAGTCTCATTTACAGGTACACTTGCA ("Noggin4-stop") and a mixture of two forward primers taken in ratio of 1:10 pM, respectively (Myc-tag-encoding sequences are underlined):<br>5'- GAGCAGAAACTCATCTCTGAAGAGGATCTGGATCTCCAGCACTATAATCAG ("Myc-Noggin4 forward") and<br>5'-GAACAGAAGCTGATATCGGAGGAAGACCTCGAGCAGAAACTCATCTCTG ("Myc forward").<br>**3d step.** Obtaining of full-length cDNA encoding *Noggin4* with target site for Noggin4 MO at 5'-UTR and three copies of Myc-tag epitope behind signal peptide cleavage site. The overlapping cDNA fragments obtained at the previous steps were purified, mixed, denatured, annealed and subjected to PCR with "Noggin4 5'-UTR" and "Noggin4-stop" primers (see above).<br>**4th step.** Cloning the PCR product into pCS2 plasmid using BamHI and XhoI sites.<br>**Final construct:** pCS2-5'-UTR-MycNoggin4.

EGFP-Noggin4 (actual mRNA: SignalNog2-3Myc-EGFP-TEV-6His-Nog4)	**1st step.** Cloning of cDNA encoding the signal peptide of *Noggin2* with 3Myc-tags into pCS2 plasmid. This cDNA fragment was obtained by PCR from MycNoggin2 plasmid (Eroshkin et al, 2006) with the following primers (here and below restriction sites are underlined): forward primer Noggin2-BamHI -<br>5'-AATTGGATCCGCCACCATGAAGAGGATAAATCTGC and<br>reverse primer Noggin2-myc BglII:<br>5'-AATTAGATCTTCTAAGCCTGAGCAGATCCT<br>and was cloned into BamHI and EcoRI (blunted) sites of pCS2 plasmid<br>Final construct: pCS2-SignalNog2-3Myc<br>**2nd step.** Cloning of EGFP cDNA into pCS2-SignalNog2-3Myc.<br>EGFP cDNA was obtained by PCR from pEGFP-C1 (Clontech) with the following primers:<br>forward EGFP-BglII: 5'-TTCTAGATCTATGGTGAGCAAGGGCGAGGA<br>and reverse EGFP-HindIII: 5'-GAAAAGCTTTGCCGCCCTTGTACAGCTCGTCCATGCC<br>and cloned into BglII and HindIII sites of pCS2-SignalNog2-3Myc plasmid.<br>Final construct: pCS2-SygnalNog2-3Myc-EGFP.<br>**3d step.** Cloning of cDNA encoding the site for TEV protease into pCS2-SignalNog2-3Myc-EGFP. cDNA of TEV site was obtained by annealing of the following oligonucleotides:<br>forward TEV-HindIII-ApaI: 5'-AGCTTTCGAAAATTTATATTTTCAGGGCCCC<br>and reverse TEV-HindIII-ApaI: 5'-AGCTGGGGCCCTGAAAATATAAATTTTCGAA<br>and cloned into HindIII site of pCS2-SignalNog2-3Myc-EGFP. Clones with the correct orientation of TEV insert were selected by PCR.<br>Final construct: pCS2-SignalNog2-3Myc-EGFP-TEV.<br>**4th step.** Cloning of 6His cDNA into pCS2-SygnalNog2-3Myc-EGFP-TEV. cDNA encoding 6His was obtained by annealing of the following oligonucleotides:<br>forward HindIII-6His: 5'-AGCTAGATCTCACCATCACCACCATCATGG<br>and reverse HindIII-6His: 5'-AGCTCCATGATGGTGGTGATGGTGAGATCT<br>and cloned into the unique HindIII site of pCS2-SignalNog2-3Myc-EGFP-TEV plasmid. Clones with the correct orientation of 6His insert were selected by PCR.<br>Final construct: pCS2-SignalNog2-3Myc-EGFP-TEV-6His.<br>**5th step.** Cloning of the mature Noggin4 cDNA into pCS2-SignalNog2-3Myc-EGFP-TEV-6His. cDNA encoding the mature Noggin4 was obtained by PCR from pBluescript-Noggin4 plasmid (Eroshkin et al, 2006) with the following primers:<br>forward Noggin4-ApaI: 5'-TTTCAGGGCCCCGGCCAAACAAATGATCTCC<br>and Stop-Noggin4-XhoI: 5'-TTACTCGAGTCAGCAGGAACACTTGCAC<br>and cloned into ApaI and XhoI sites of pCS2-SignalNog2-3Myc-EGFP-TEV-6His.<br>Final construct: pCS2-SygnalNog2-3Myc-EGFP-TEV-6His-Nog4.
EGFP-Noggin1 (actual mRNA: SignalNog2-3Myc-EGFP-TEV-6His-Nog1)	cDNA of Noggin4 in pCS2-SygnalNog2-3Myc-EGFP-TEV-6His-Nog4 was swapped with Noggin1 cDNA obtained by PCR from pBluescript-Noggin1 plasmid[2] with the following primers (here and below the stop codon is framed):<br>forward Noggin1-ApaI: 5'-TTTCAGGGCCCCCATTATCTGCACATCAGAC<br>and Stop-Noggin1-XhoI: 5'- ATTCTCGAGTC TCA GCATGAGCATTTGCA.
EGFP-Noggin2 (actual mRNA: SignalNog2-3Myc-EGFP-TEV-6His-Nog2)	cDNA of Noggin4 in pCS2-SignalNog2-3Myc-EGFP-TEV-6His-Nog4 was swapped with Noggin2 cDNA obtained by PCR from pBluescript-Noggin2 plasmid[2] with the following primers:<br>forward Noggin2-ApaI: 5'-TTTCAGGGCCCCCAGCCTTATCTCAGGCTTA<br>and Stop-Noggin2-XhoI: 5'- ATTCTCGAG TTA GCATGAACACTTACACTCTG.
EGFP-XWnt8 (actual mRNA: SignalNog2-3Myc-EGFP-TEV-6His-XWnt8)	cDNA of Noggin4 in pCS2-SignalNog2-3Myc-EGFP-TEV-6His-Nog4 was swapped with XWnt8 cDNA obtained by PCR with the following primers:<br>forward XWnt8-ApaI: 5'- TTTCAGGGCCCCTGGTCAGTCAATAACTTTC<br>and Stop-XWnt8-XhoI: 5'- TAATCTCGAG TCA TCTCCGGTGGCCTCTGT.

EGFP-hep (actual mRNA: SignalNog2-3Myc-EGFP-TEV-6His-hep)	The following oligonucleotides encoding the heparin-binding motif of *Noggin1* were annealed and swapped with Noggin4 cDNA in pCS2-SignalNog2-3Myc-EGFP-TEV-6His-Nog4:<br>forward Heparin-ApaI-XhoI:<br>5'-AGAGCAAAAAGCACAGACTGAGCAAGAAACTCAGGAGAAAGTGAC<br>and reverse Heparin-ApaI-XhoI:<br>5'-TCGAGTCACTTTCTCCTGAGTTTCTTGCTCAGTCTGTGCTTTTTGCTCTGGCC.
TagRFP-Noggin1,2 and 4 (actual mRNAs: SignalNog2-3Myc-TagRFP-TEV-6His-Nog1, 2 or 4)	cDNA of EGFP in pCS2-SignalNog2-3Myc-EGFP-TEV-6His-Nog1, 2 and 4 was swapped with cDNA of TagRFP obtained by PCR with the following primers:<br>forward TagRFP-BglII: 5'-TTCTAGATCTATGGTGTCTAAGGGCGAAGA<br>and reverse TagRFP-BglII: 5'-TTCTAGATCTCCAATTAAGTTTGTGCCCCA.
TagRFP-XWnt8 (actual mRNA: SignalNog2-3Myc-TagRFP-TEV-6His-XWnt8)	cDNA of EGFP was swapped in pCS2-SignalNog2-3Myc-EGFP-TEV-6His-Nog4 with cDNA of XWnt8 obtained by PCR with the following primers:<br>Forward XWnt8-ApaI: 5'- TTTCAGGGCCCCTGGTCAGTCAATAACTTTC<br>and Stop-XWnt8-XhoI: 5'- TAATCTCGAG TCA TCTCCGGTGGCCTCTGT.
TagRFP-hep (actual mRNA: SignalNog2-3Myc-TagRFP-TEV-6His-hep)	cDNA of EGFP in pCS2-SignalNog2-3Myc-EGFP-hep was swapped with cDNA of TagRFP by using flanking BglII sites.
pCMV-3Myc-6His-Nog4 (suitable for neomycin selection)	The cassette SignalNog2-3Myc-EGFP-TEV-6His-Nog4 was excised from pCS2-SignalNog2-3Myc-EGFP-TEV-6His-Nog4 plasmid (see above) with BamHI and XhoI restrictases and sub-cloned into BglII and XhoI sites of pEGFP-N1 vector (Clontech). Then, EGFP cDNA was removed from SignalNog2-3Myc-EGFP-TEV-6His-Nog4 cassette by cutting at BglII sites, and the linearized plasmid was self-ligated. At the final step, the second EGFP cDNA was removed from the plasmid by cutting at BamHI and NotI sites, the linearized plasmid was treated by Klenow fragment and self-ligated.

Table S1. Morpholino oligonucleotides, PCR primers and cloning strategy. All DNA constructs were checked by sequencing.

Movie S1. Comparison of EGFP-Noggin4 and EGFP-Noggin2 FRAP dynamics in the intercellular space of the animal ectoderm of the *Xenopus laevis* early gastrula. Intercellular spaces (IS) with EGFP-Noggin4 and EGFP-Noggin2 are shown. Bleached regions are visualised by white squares (15×15 and 7.5×7.5, respectively). In the case of EGFP-Noggin4, broader area was bleached purposely to make its quick diffusion more evident. Fluorescence recovery is demonstrated both by microscopy imaging and by normalised FRAP curves. A violet vertical line on every plot is moving in correspondence with the upper photo changes.

Movie S2. FRAP experiments demonstrating retardation of EGFP-Noggin4 diffusion in the presence of TagRFP-Wnt8. Intercellular spaces containing EGFP-Noggin4 alone (upper left) and in the presence of TagRFP-Wnt8 (upper right) are shown. The presence of TagRFP-Wnt8 within IS on the upper right panel is shown in the red channel during a short time interval before bleaching. Bleached regions are visualised by white squares (7.5×7.5 μm). Fluorescence recovery of EGFP-Noggin4 is demonstrated both by microscopy imaging in the green channel and by normalised FRAP curves. A violet vertical line on every plot is moving in correspondence with the upper photo changes.

a.	Weight at WB, *ng*	Dilution ratio	Total weight, *ng*	IP concentration, μM
Bound #1	44.3	0.5	88.7	0.0059
Unbound #1	195.4	0.025	7817.0	0.5211
Bound #2	28.6	0.5	57.2	0.0038
Unbound #2	89.7	0.05	1793.3	0.1196
Bound #3	24.6	0.5	49.2	0.0033
Unbound #3	111.2	0.1	1112.3	0.0742
Bound #4	7.8	0.5	15.6	0.0010
Unbound #4	85.1	0.2	425.5	0.0284
Bound #5	2.0	0.5	4.0	0.0003
Unbound #5	70.3	0.2	351.6	0.0234

b.	Weight at WB, *ng*	Dilution ratio	Total weight, *ng*	IP concentration, μM
Bound #1	36.4	0.2	182.06	0.0121
Unbound #1	54.2	0.0125	4334.84	0.2890
Bound #2	54.9	0.5	109.7	0.0073
Unbound #2	68.8	0.025	2753.7	0.1836
Bound #3	73.1	0.5	146.1	0.0097
Unbound #3	68.1	0.05	1362.6	0.0908
Bound #4	49.7	0.5	99.5	0.0066
Unbound #4	133.7	0.1	1337.4	0.0892
Bound #5	40.9	0.5	81.8	0.0055
Unbound #5	80.2	0.2	401.2	0.0267
Bound #6	2.1	0.5	4.1	0.0003
Unbound #6	40.2	0.2	201.0	0.0134

Table S2. Calculation of the amount of Flag-Wnt8 for quantitative co-immunoprecipitation. **(a)** Precipitation of Flag-Wnt8 with the pure anti-Myc resin. **(b)** Precipitation of Flag-Wnt8 with the anti-Myc resin conjugated to Myc-Noggin4. In the first column we present weights calculated according to image analysis of Western and Flag-BAP calibration (Fig. S6a). Next, total weights were calculated taking into account the dilution ratio (column 2). At last the concentration in the precipitation reaction volume was computed taking into account the IP volume (300 μl) and Wnt8 molar weight. The adsorption isotherm at Fig. S6b was plotted using final concentrations in the right column.

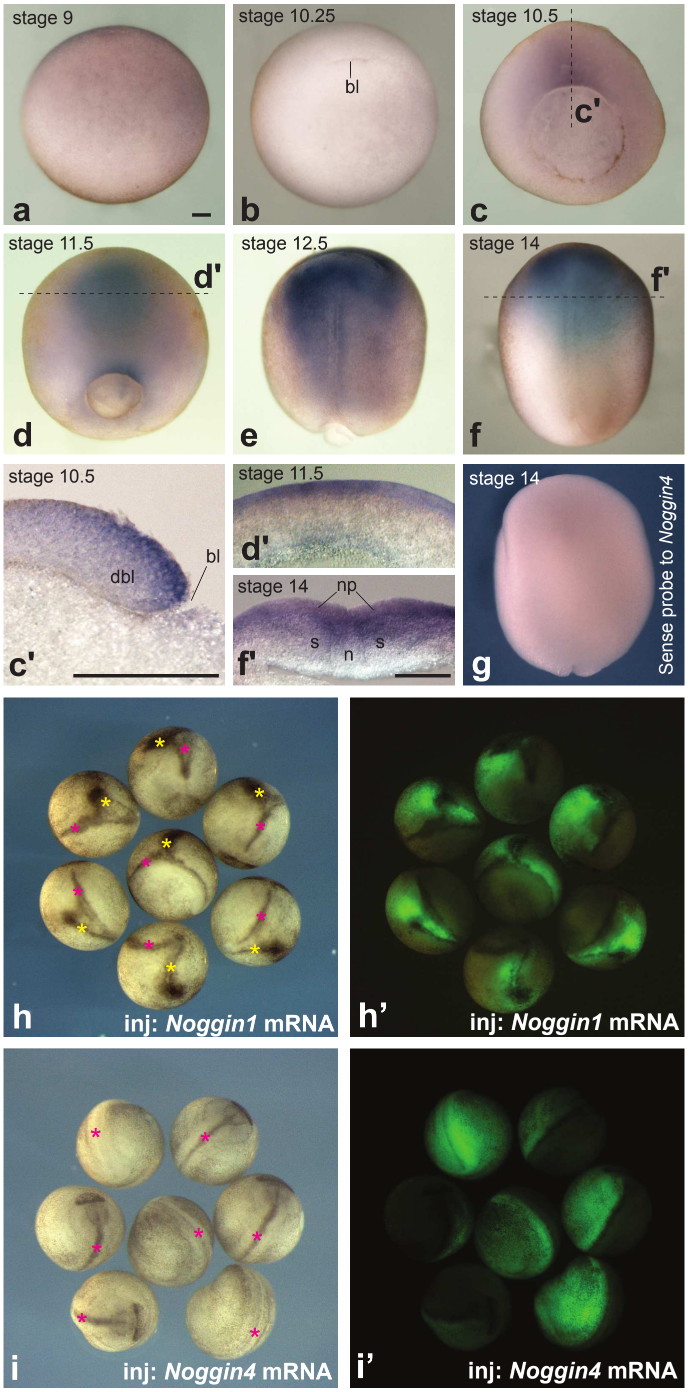

Figure S1. Analysis of the expression pattern of *Noggin4* and its ability to induce secondary axes. (a-f) The expression pattern of *Noggin4* on successive stages, from the late blastula (stage 9) till early-midneurulas (stage 14), as revealed by the whole-mount *in situ* hybridisation. Note, that at all these stages, a maximum of the expression is located within the presumptive anterior neural plate. All embryos are shown from the vegetal pole, anterior to the top. (**c'–f'**) Vibratom sections of embryos at stage 10.5, 11.5 and 14 made as indicated on (**c** and **e**) by dashed lines. (**g**) Control *in situ* hybridization with sense probe to *Noggin4* mRNA. (**h** and **h'**) Injection of 15 ng/blastomere of *Noggin1* mRNA mixed with FLD, into the equatorial zone of ventral blastomeres at 4-cell stage induces formation of secondary body axes (98%, n=120; red asterisk — main axis; yellow asterisk — secondary axis). (**i** and **i'**) By contrast, injections by the same way of even 150 ng/blastomere of *Noggin4* mRNA did not induce secondary axes at all (0%, n=120; asterisk — main axis).Abbreviations: bl — blastopore, dbl — dorsal blastopore lip, n — notochord, np — neural plate, s — somite. Bar everywhere is 100 μ.

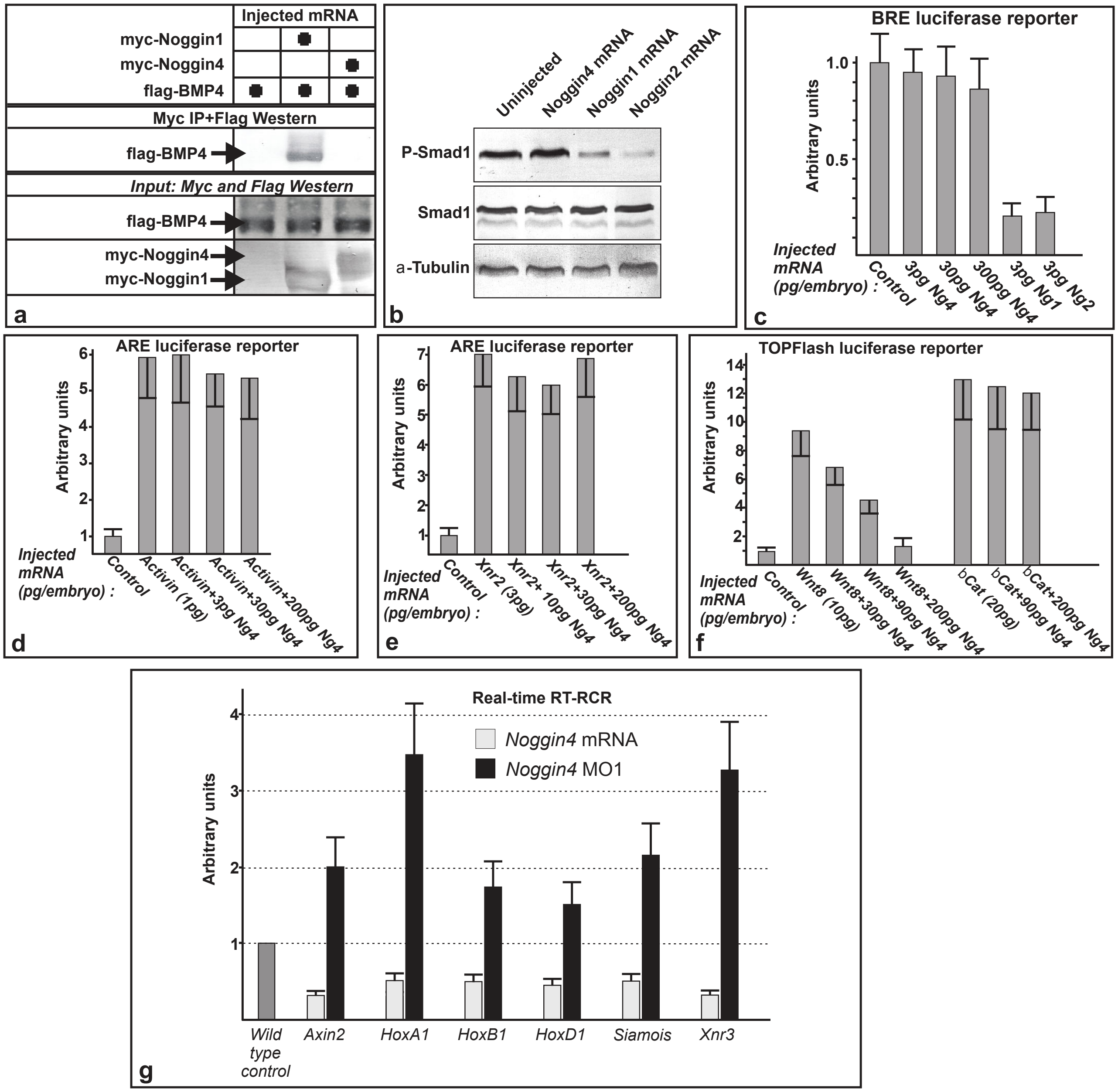

Figure S2. Noggin4 cannot antagonise BMP and Activin/Nodal signalling but inhibits Wnt/β-Catenin pathway. (a) Comparison of Noggin1 and Noggin4 abilities to bind BMP4, using immunoprecipitation. **(b)** In contrast to Noggin1 and 2, Noggin4 does not cause reduction of endogenous phosphorylation of Smad1. Embryos were injected with *Noggin1/2* or *Noggin4* mRNA (100pg/embryo) and phosphoSmad1 was detected on Western-blot by anti-phosphoSmad1 antibody (Cell Signaling Technology, Inc.). Overall level of Smad1 was detected by anti-Smad antibodies (ab66737, Abcam). Alpha-tubulin serving as a loading control was revealed by anti-tubulin antibodies (DM1A, Sigma). In all these cases, Fab fagments of Anti-Rabbit antibodies conjugated to alkaline-phospotase (A3937, Sigma) were used for detection of primary antibodies. **(c)** Effects of Noggin1, Noggin2 and Noggin4 on expression of the BMP-specific luciferase reporter, BRE. Embryos at two-cell stage were injected with BRE plasmid, either alone (control) or mixed with *Noggin1* or *Noggin2* mRNAs taken in a fixed concentration, or *Noggin4* mRNA taken in increasing concentrations. (**d** and **e**) Effects of Noggin4 on expression of the Smad2-specific reporter ARE activated by ActivinB or Xnr2. **(f)** The effect of Noggin4 on expression of the Wnt/β-Catenin-specific reporter TOPFlash activated by Wnt8 and β-Catenin . **(g)** qRT-PCR analysis of expression of the direct genetic targets of the canonical Wnt pathway: *Axin2*, *HoxA1*, *HoxB1*, *HoxD1*, *Siamois* and *Xnr3*, in the late gastrula embryos (stage 12) injected at 4-cell stage with *Noggin4* mRNA or *Noggin4* MO1. For each of the tested genes, the expression level in the wild-type embryos was taken as one unit.

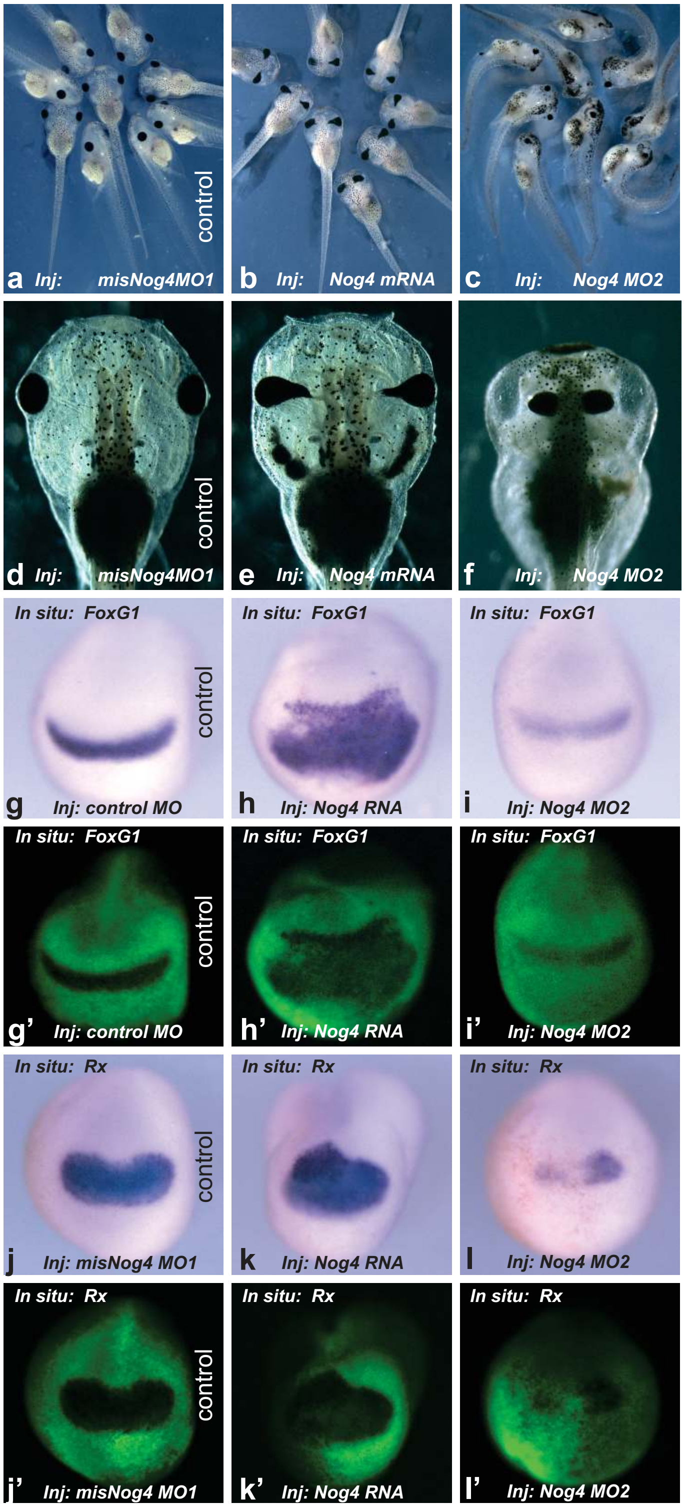

Figure S3. Effects of *Noggin4* mRNA and MO2 injections on tadpole head structures and expression of anterior markers, *FoxG1* and *Rx*, at the midneurula stage. **(a–c)** 5 days tadpoles injected as indicated. **(d–f)** Heads of 5 days tadpoles injected as indicated are shown from the dorsal side, anterior to the top. **(g-l')** *In situ* hybridisation with dig-probes to *FoxG1* and *Rx* mRNA of midneurula embryos injected with *control* MO provided by GeneTools (100%, n=30 (g and g')), *Noggin4* mRNA (85%, n=30 (h and h') and 61%, n=35 (k and k')), *misNoggin4* MO2 (100%, n=40 (i and i', l and l')), and *misNoggin4* MO1 (100%, n=40 (f) and 80%, n=40 (i)).

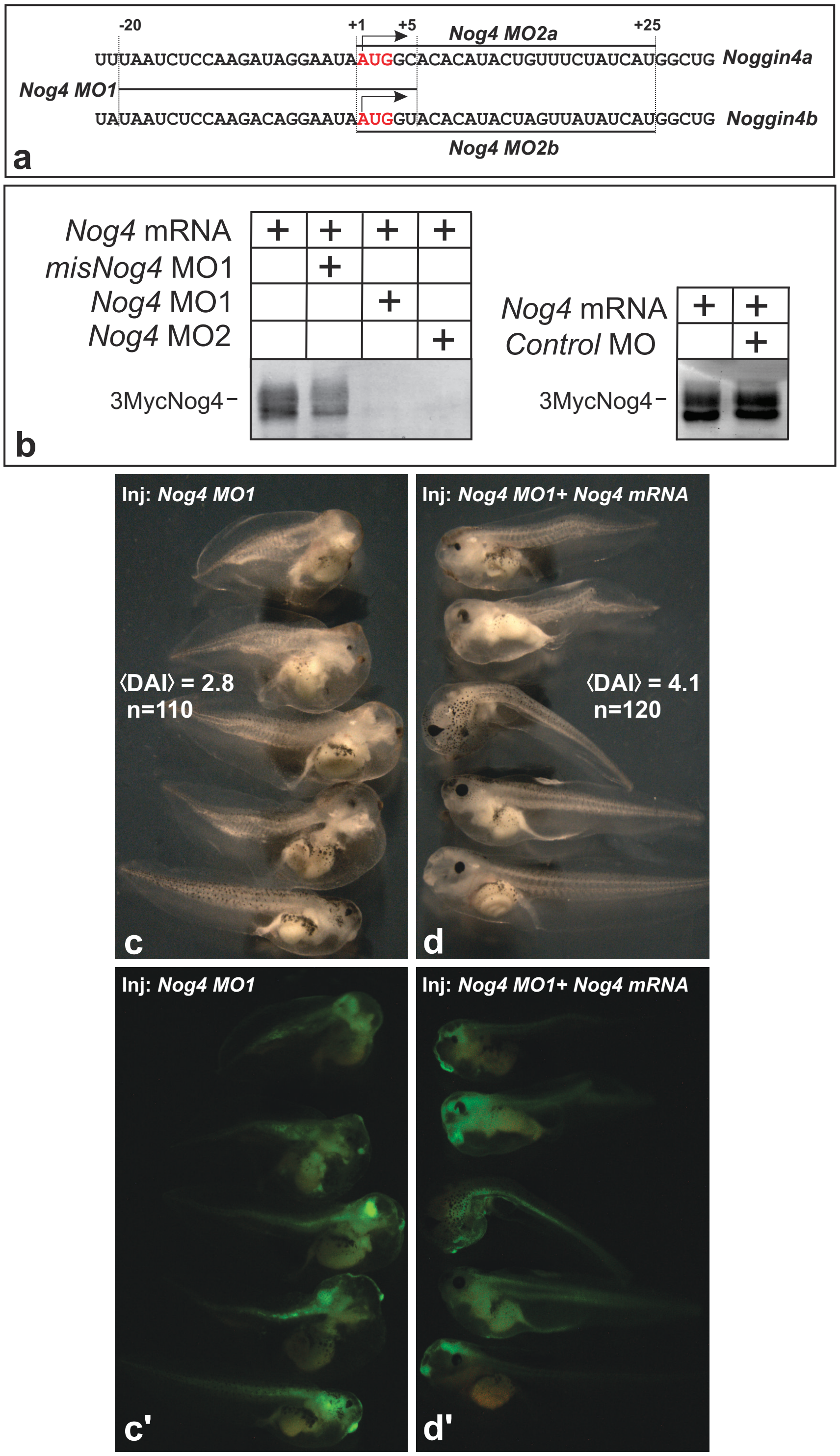

Figure S4. Testing of MO specificity and efficiency. **(a)** Schema of the morpholino target sites location on both pseudoalleles of the *Xenopus laevis Noggin4* mRNA. **(b)** 5'-UTR-Myc-Noggin4 mRNA were injected into each blastomere of 2-cell *Xenopus laevis* embryos (100 pg/blastomere), either alone or in a mixture with control *misNoggin4 MO*, *MO1*, *MO2* (8 nl of 0.2 mM water solution) or the standard control MO provided by GeneTools (8 nl of 0.5 mM water solution). The injected embryos were collected at the midgastrula stage and analysed for presence of Myc-Noggin4 by Western blotting with anti-Myc antibody (see Materials and Methods for details). **(c-d')** The specificity of the MO effects was confirmed by rescue experiments, in which *Noggin4 MO1* was injected either alone **(c and c')** or with *Noggin4* mRNA deprived of the MO1 target site **(d and d')**. Medial Dorso-Anterior Index (DAI) was calculated for both types of tadpoles developed from the injected embryos.

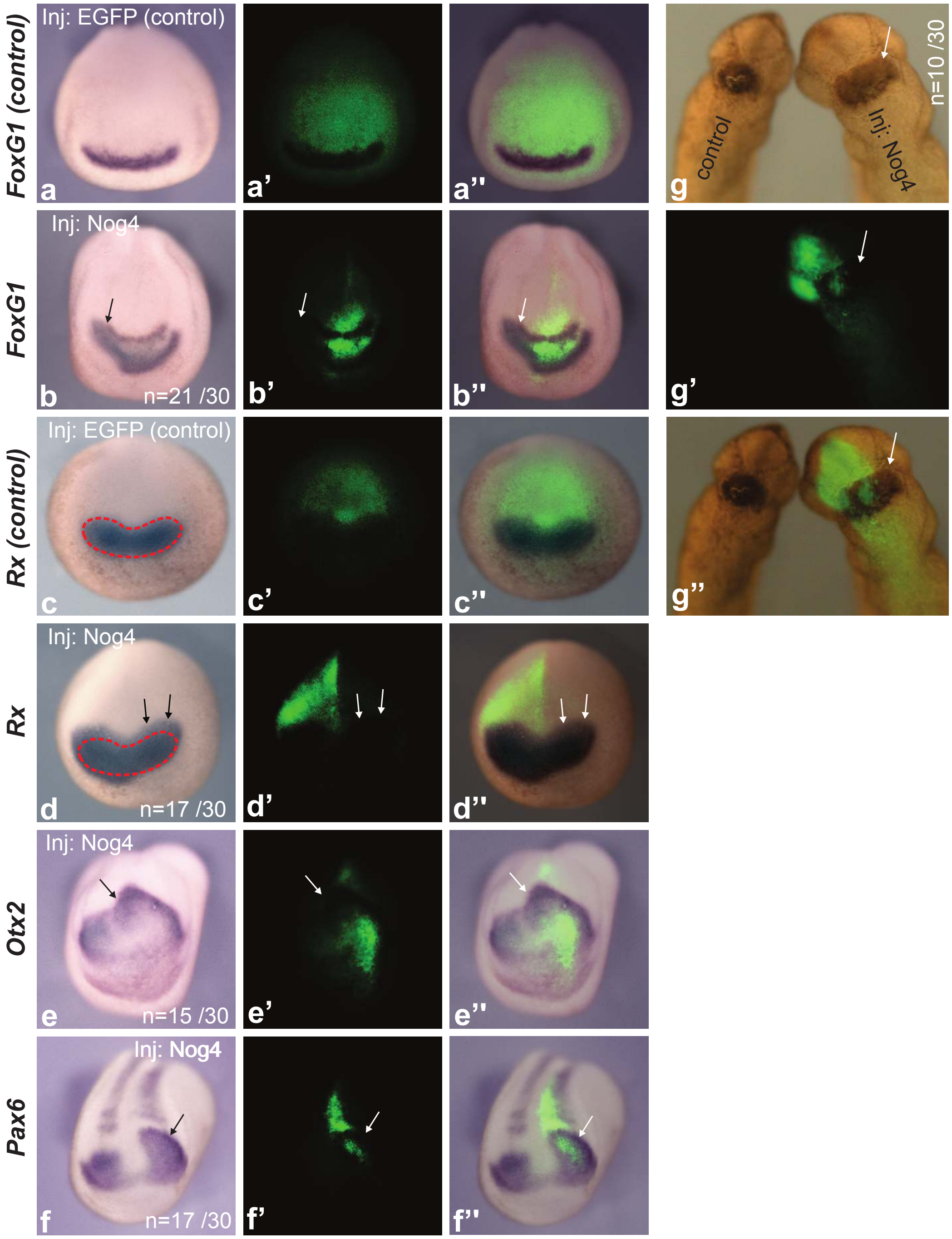

Figure S5. Noggin4 acts cell-non-autonomously when promoting the expression of anterior markers. (a) Control embryo injected in dorsal blastomeres with EGFP mRNA mixed with FLD tracer at 8-cell stage and hybridised at the midneurula stage with FoxG1 probe. This embryo is presented for comparison of width of the control *FoxG1* expression band with width of *FoxG1* expression band in the embryo injected with *Noggin4* mRNA and shown on **b**. **(b-f)** Midneurula embryos injected with *Noggin4* mRNA mixed with FLD tracer unilaterally, in one of the animal dorsal blastomeres, at 8-cell stage and hybridised with probes to the indicated marker genes. Arrows indicate areas in which overexpression of the tested marker genes does not match the localisation of FLD, thereby confirming cell-non-autonomous activity of Noggin4. **(a'-f')** Fluorescent images of the same embryos as on **a–f**. **(a''–f'')** Superimposed images of **a–f** and **a'–f'**. **(g–g'')**. An expanded cement gland on the non-injected side of the tail-bud stage embryo (right embryo), which was injected in the right animal dorsal and ventral blastomeres at 8-cell stage, confirms cell-non-autonomous activity of Noggin4.

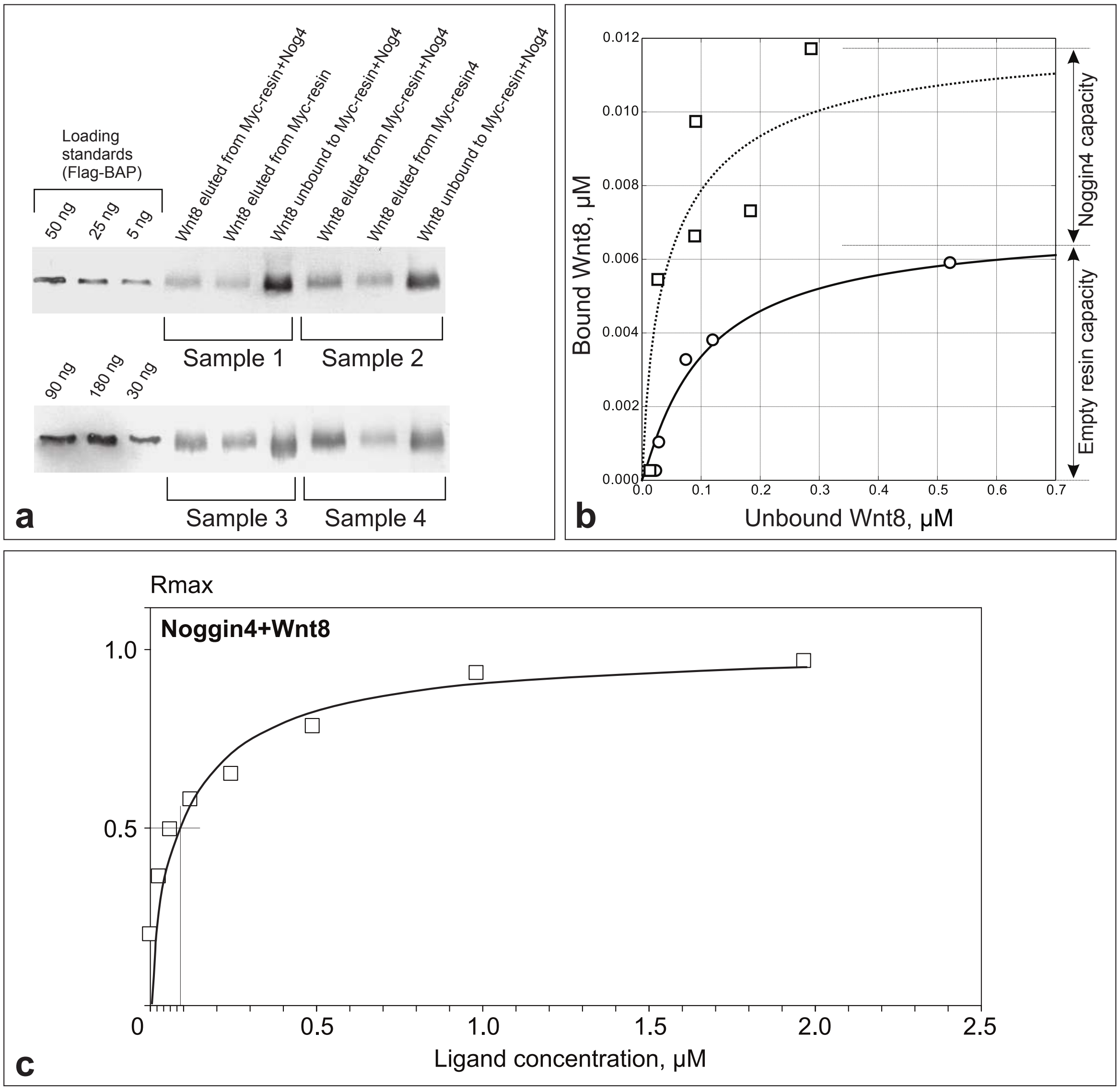

Figure S6. ***In vitro* analysis of Wnt8 binding to Noggin4 by quantitative co-immunoprecipitation and Surface Plasmon Resonance assay. (a)** Representative examples of Western blotting (WB) with antiFlag-antibody of indicated amounts of Flag-BAP (loading standard), Flag-Wnt8 eluted by Myc-peptide after incubation with Myc-Resin+Myc-Noggin4, Flag-Wnt8 eluted by Myc-peptide after incubation with Myc-resin+control samples and unbound Flag-Wnt8 after incubation with Myc-Resin+Myc-Noggin4. Loading standards and experimental samples were put on the same gels. **(b)** Adsorption isotherms drawn over experimental points (Table S2) obtained from WB images. Theoretical fits based on equations **(3)** and **(4)** are depicted with solid and dashed lines, respectively. Parameter values are provided in Materials and Methods. **(c)** Titration curves obtained from SPR measurements for interaction between immobilised Noggin4 and Wnt8.

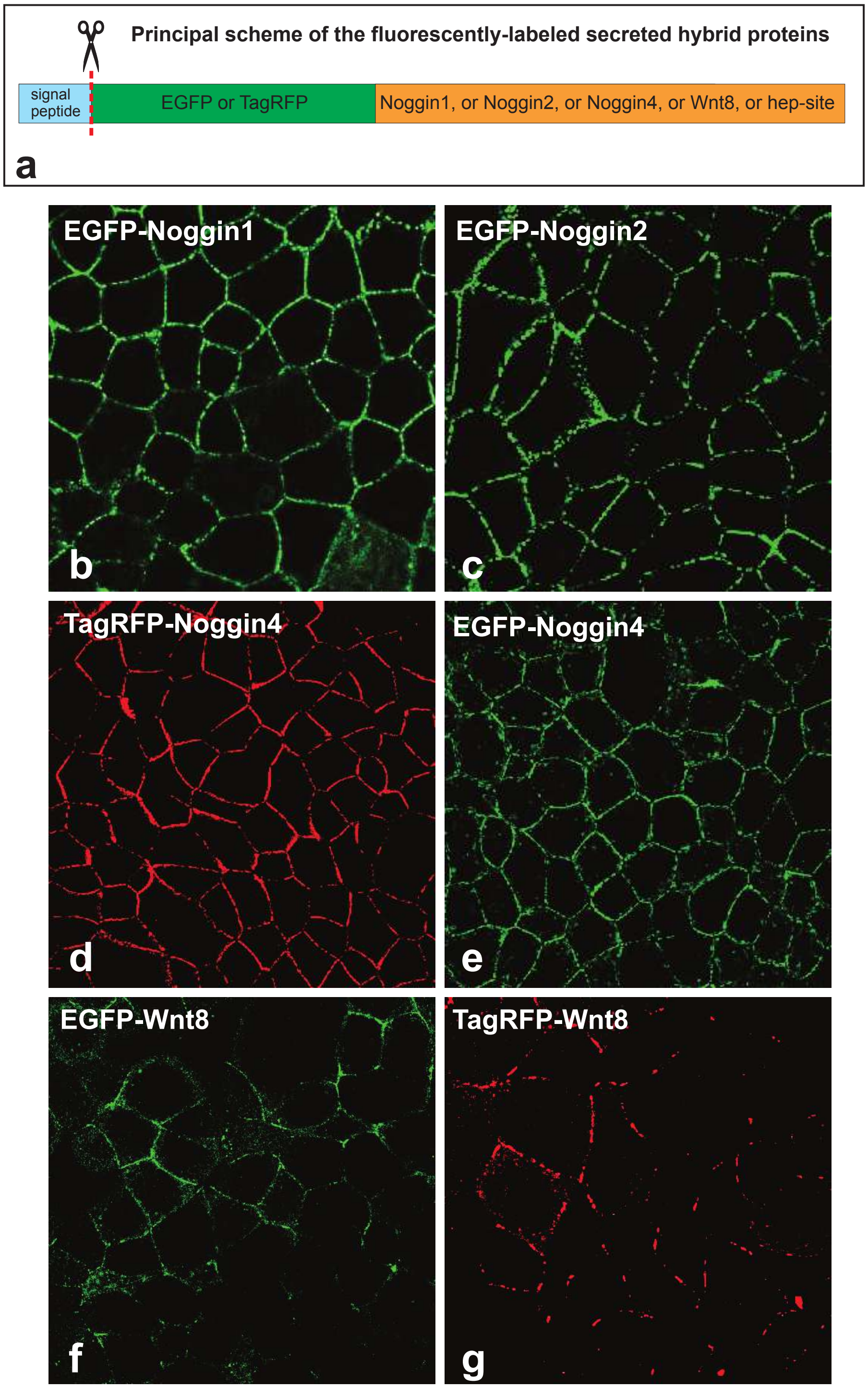

Figure S7. **Noggin1, Noggin2, Noggin4 and Wnt8 fused to EGFP or TagRFP are secreted into the intracellular space of animal ectoderm of the *Xenopus laevis* embryos.** **(a)** Principal scheme of the fluorescently-labeled secreted proteins. **(b-g)** Visualisation of the indicated fusion proteins in the intercellular space of the animal ectoderm of living *Xenopus laevis* embryos. Embryos at 4-cell stage were injected into animal blastomeres with mRNAs encoding indicated fusion proteins. At early-midgastruala stage, the injected embryos were photographed from the animal pole using the confocal microscope.

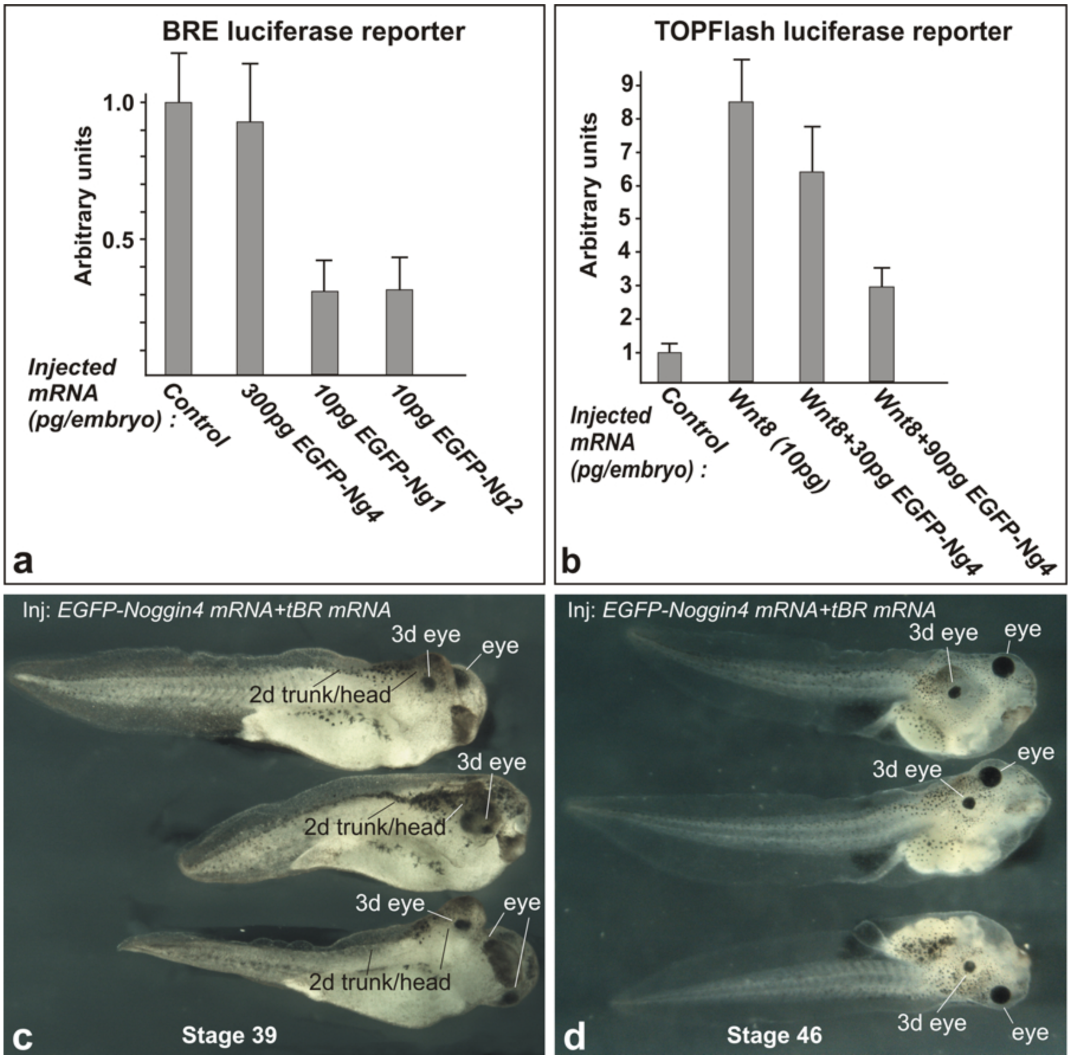

Figure S8. **Fusions of Noggin1, Noggin2 and Noggin4 with EGFP retain their molecular and physiological activities.** (**a**) EGFP-Noggin1 and EGFP-Noggin2 inhibit expression of the BMP-specific luciferase reporter, BRE, in *Xenopus laevis* embryos. The embryos were injected with BRE reporter plasmid, either alone (control) or mixed with *EGFP-Noggin1* or *EGFP-Noggin2* mRNAs. (**b**) EGFP-Noggin4 inhibits expression of the Wnt/β-Catenin-specific luciferase reporter, TOPFlash, in *Xenopus laevis* embryos. The embryos were injected either with TOPFlash reporter mixed with XWnt8 mRNA (control) or with TOPFlash reporter plasmid mixed with XWnt8 mRNA and increasing concentrations of EGFP-Noggin4 mRNA. (**c and d**). *EGFP-Noggin4* mRNA co-injected with mRNA of the BMP inhibitor, truncated BMP receptor tBR, induces development of secondary trunk and head structures (better visible on **c**), including cyclopic eyes, in *Xenopus laevis* tadpoles (50%, n=70). Two different sets of tadpoles (obtained in the independent experiments) were photographed at stage 39 and 46 respectively. On **c** two upper tadpoles are shown from the right side and the bottom tadpole is shown from the ventral side. On **d** two upper tadpoles are shown from the right side and the bottom tadpole from the left side.

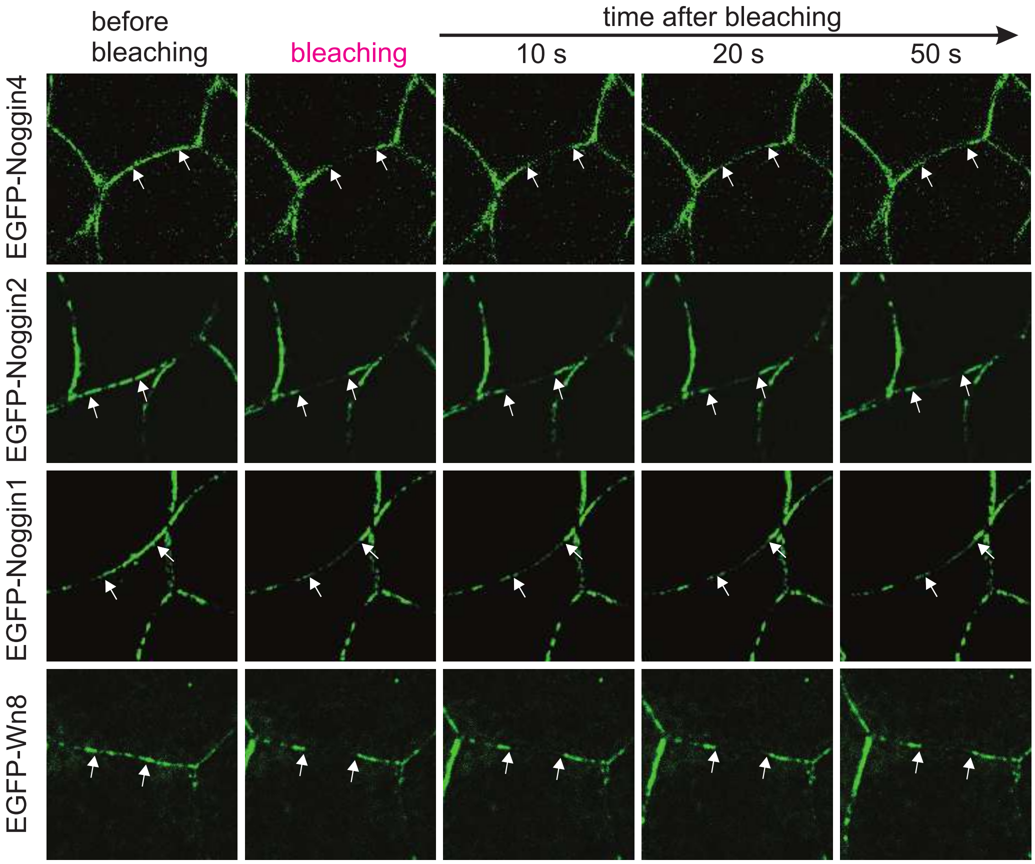

Figure S9. Representative examples of FRAP in the intracellular space (IS) of animal ectoderm of embryos that secrete the hybrid proteins indicated on the left. Arrows indicate the margins of the bleached region.

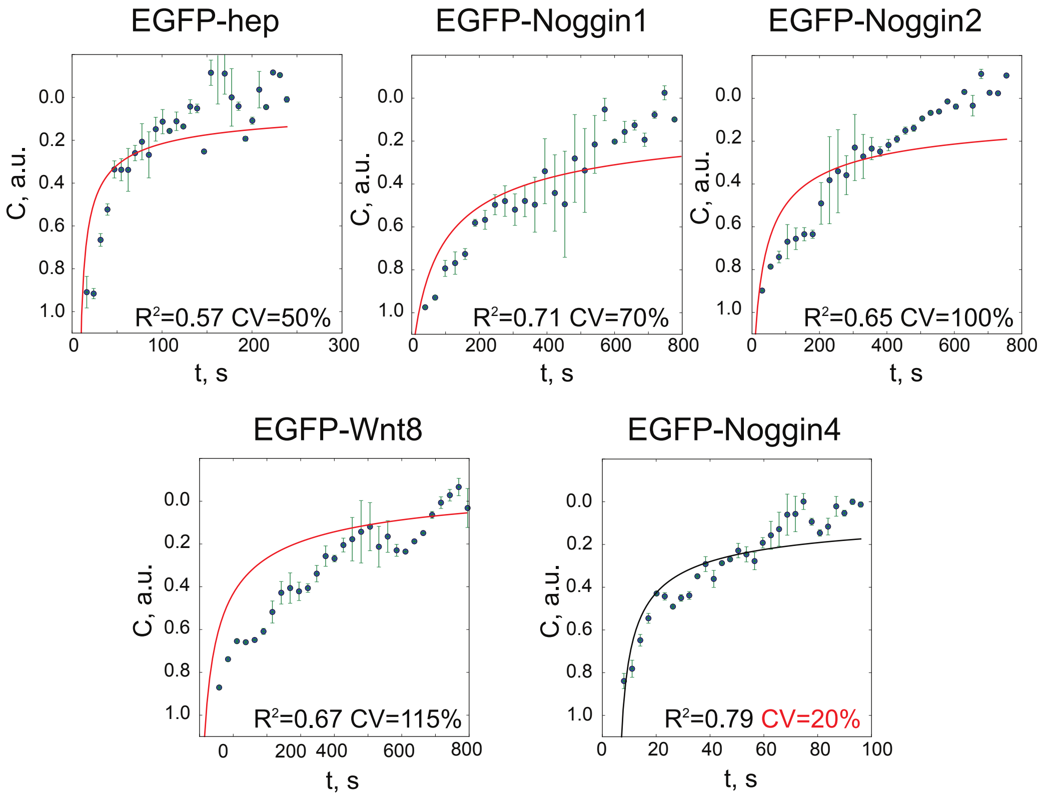

Figure S10. FRAP data fitted to the equation (S1.2). Averaged normalised data with error bars and approximation curves are plotted for every experiment. The determination coefficient (R^2) is presented for every group of observations. The lowest coefficient of variation (CV) of parameter D calculated for every separate measurement was observed for EGFP-Noggin4.

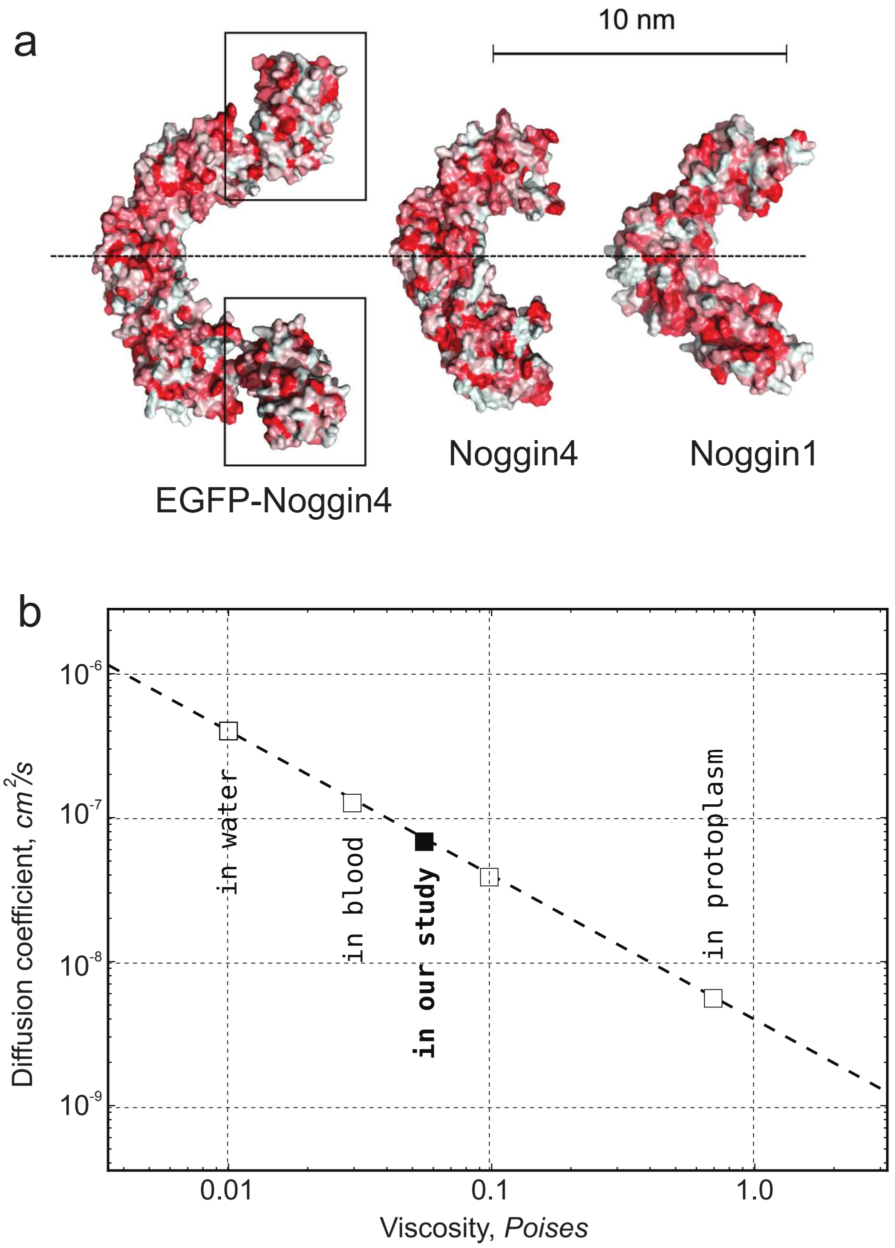

Figure S11. Hydrodynamic modelling of Noggins diffusivity. (a) Solvent accessible surfaces of Noggin1, Noggin4 and EGFP-Noggin4 molecules were predicted by homological modelling and molecular dynamic equilibration. C_2 symmetry axis (dashed line) passes through the interdimeric connection. Fused EGFP molecules are framed by rectangles. **(b)** Calculated diffusivity of the Noggin4-EGFP dimer in dependence of solvent viscosity. Viscosity values for water (at 300 K), blood[3] and protoplasm[4] were selected as calibration points.

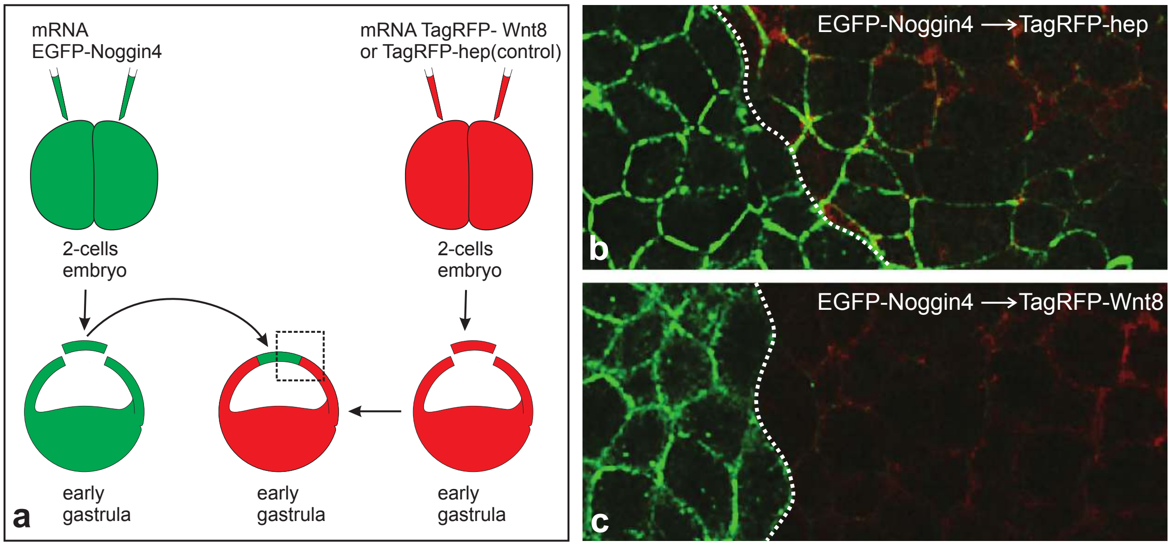

Figure S12. Investigation of Noggin4 interaction with Wnt8 via the transplantation assay. (a) Schematic of the experiment. The region shown in **b** and **c** is framed by a dashed rectangle. **(b and c)** The diffusion of EGFP-Noggin4 from the transplant spreads substantially faster in the IS of the recipient embryo that secretes TagRFP-hep **(c)** compared with the recipient embryo that secretes TagRFP-Wnt8 (**b**). In each case, the border between the transplant and the recipient tissues is marked by a white dotted line. Animal pole view.

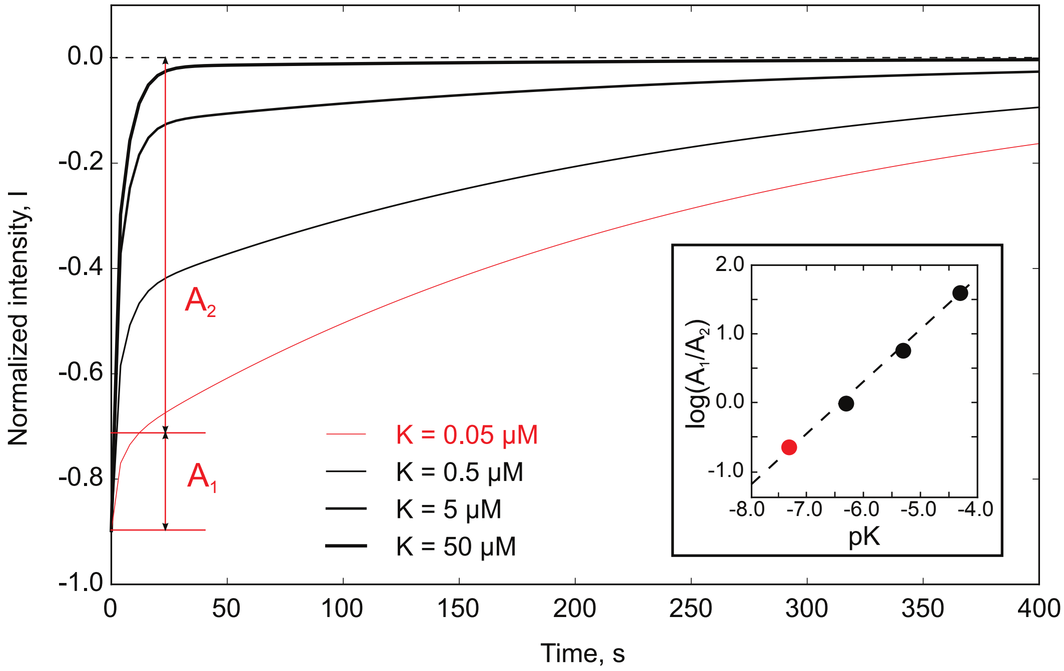

Figure S13. FRAP kinetics with different dissociation constants formed by numerical solution of the system of equations (S1.5)**.** Parameter values were set as following: $k_{off} = 1/250s^{-1}$; $l = 7.5\mu m$; $b(0) = a(0) = 1\mu M$; $D = 1\mu m^2/s$; initially bleached protein was distributed normally with dispersion equal to a half of the reactor length l. The step of the coordinate grid was l/100; integration step was 2·10−4 s. Every numerical curve was fitted to the two-exponential equation and the ratio of pre-exponential factors $A_{1,2}$ (S1.11) is shown against the dissociation constant in double-logarithmic coordinates (insert).

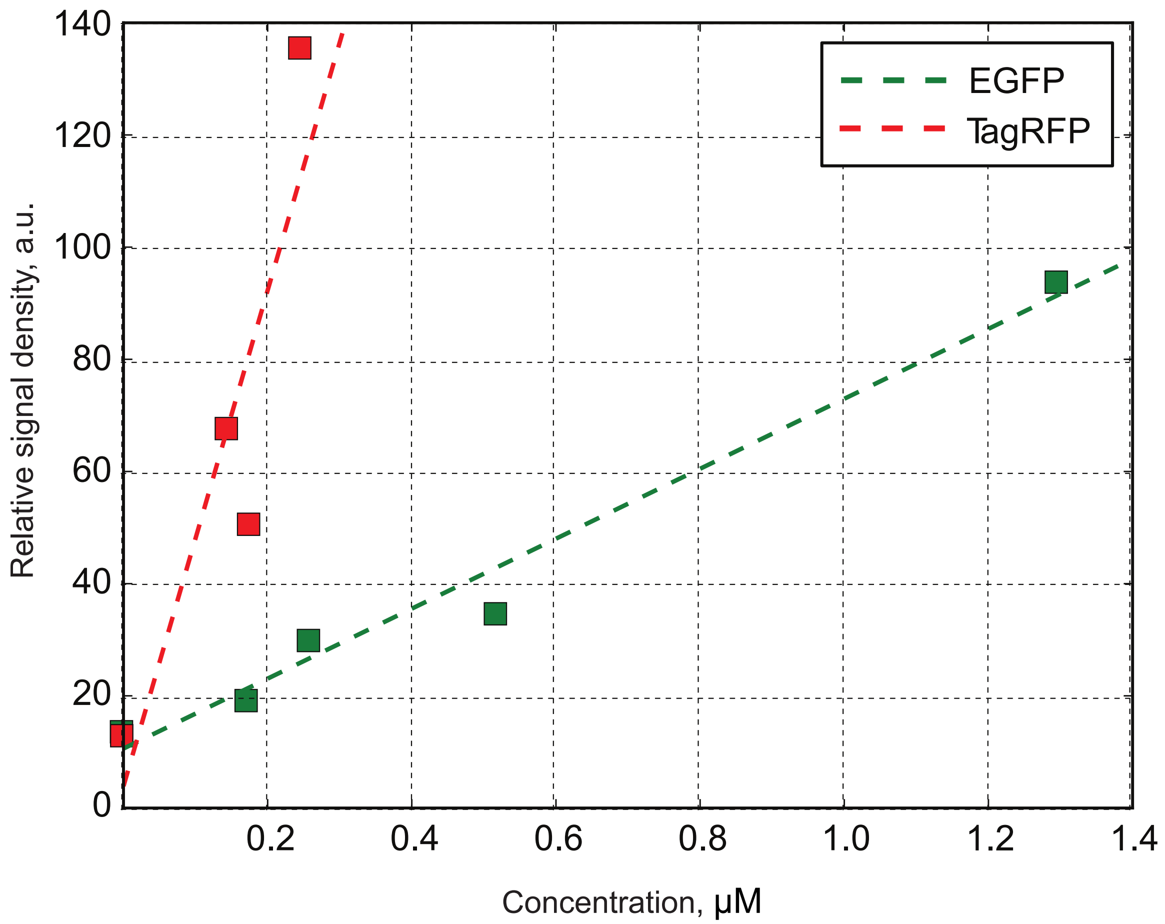

Figure S14. Calibration of the confocal microscope. Calibration curves for EGFP (green) and TagRFP (red) proteins under the most usable microscopy settings.

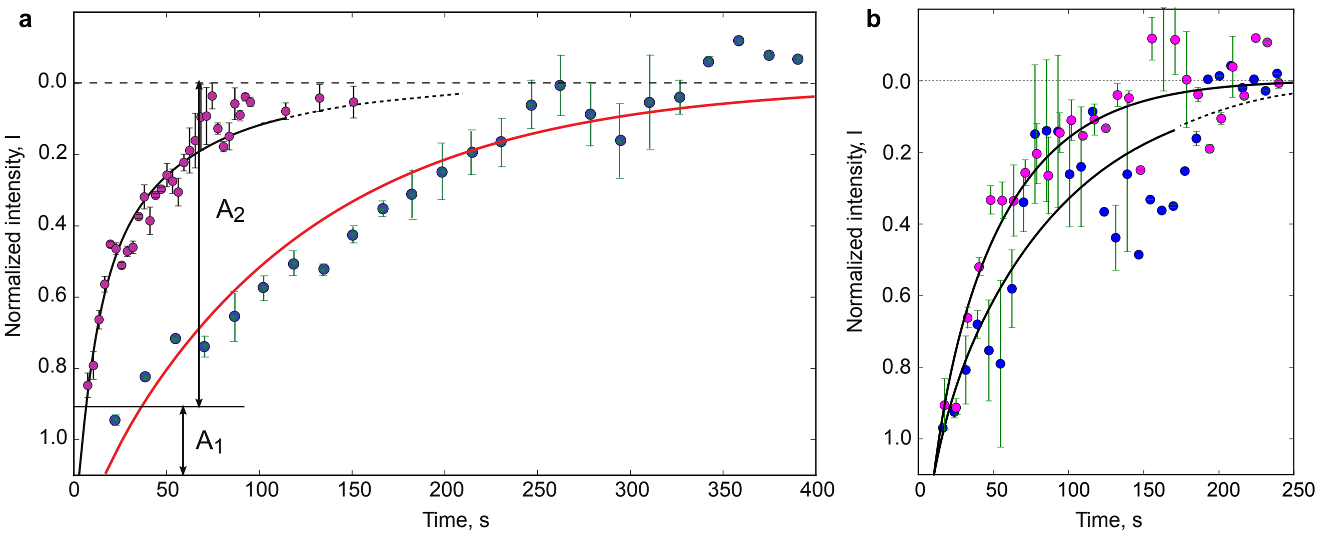

Figure S15. Diffusion retardation of EGFP-Noggin4 in the presence of TagRFP-Wnt8. FRAP curves of EGFP-Noggin4 **(a)** and EGFP-hep **(b)** were measured in the area of co-localisation with TagRFP-Wnt8 (blue circles). FRAP kinetics of EGFP-Noggin4 **(a)** and EGFP-hep **(b)** in the absence of TagRFP-Wnt8 are depicted by violet circles. Recovery kinetics of EGFP-Noggin4 and EGFP-hep in the presence of TagRFP-Wnt8 was fitted by the model described by the equation (S1.5) (Supplementary Materials and Methods, 1.2). The following parameter values were used: $k_{off} = 0.01s^{-1}$; $k_on = 10^5 M^{-1}s^{-1}$; $l = 7.5\mu m$; $[B]_0 = [A]_0 = 1\mu M$; $D = 1\mu m^2/s$; the initial distribution of both bleachable substances was $c(x) = c_0 \sin(\pi x/l)$, EGFP-Noggin4 ("A" in (S1.3) notations) or EGFP-Noggin4-Wnt8 complex ("C" in (S1.3) notations). The curves obtained in the absence of TagRFP-Wnt8 were fitted using the two-exponential kinetics (S1.11).

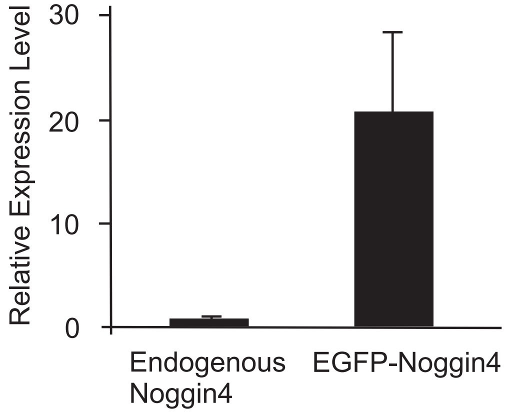

Figure S16. Relative concentrations of the endogenous Noggin4 and EGFP-Noggin4 mRNAs in the ectodermal explants of the midgastrula stage embryos. 70 pg of *EGFP-Noggin4* mRNA was injected into the animal pole cytoplasm of each dorsal blastomere of 4-cell stage embryos. The dorsal ectoderm explants were excised and collected from injected and wild-type, non-injected, sibling embryos at stage 11, followed by RNA extraction. qRT-PCR with primers for Noggin4 and EGFP was performed for samples obtained from non-injected and injected embryos, respectively. The data were normalised relative to expression of two housekeeping genes, *ODC* and *EF1*α as described in.[5] The expression level of the endogenous Noggin4 in the wild-type samples was used for normalisation and regarded as one unit.

Supplementary Materials and Methods

1. Mathematical analysis of FRAP data

1.1. Calculation of the free diffusion coefficient on the basis of FRAP curves

The advantage of the *Xenopus* embryo model is that it allows to measure FRAP kinetics in single intercellular spaces between large flat ectodermal cells, avoiding technical complications. This model allows us to consider diffusion as a one-dimensional process, and record FRAP kinetics in a comparatively short time: less than 1 min. in the case of Noggin4. Accordingly, these characteristics of the *Xenopus* embryo model allowed us to use the simple equation of one-dimensional FRAP model described by Ellenberg et al.[6] The exact solution of this equation has a complex form and contains special functions. However, for most practical purposes, the following simple empirical equation that differs from the exact solution by less than 5%, can be used instead of the exact equation:[6]

$$\frac{I_0 - I_t}{I_m - I_0} = \frac{I_0 - I_\infty}{I_m - I_0}\left(1 - \left(\frac{4\pi Dt}{w^2} + 1\right)^{-1/2}\right) \tag{S1.1}$$

where I_0 is the background value of the fluorescence intensity (FI) in the middle point of a bleached region; I_m is the background value of FI outside of the intercellular spaces; $I(t)$ is a FI value in the middle-point at time t; w is the length of the bleached region; $I(\infty)$ is the FI value in the middle-point after complete recovery. If we denote the normalised concentration of the bleached substance by C_z, then the equation (S1.1) transforms to:

$$\frac{I(t) - I(\infty)}{I_0 - I(\infty)} = \left(\frac{4\pi Dt}{w^2} + 1\right)^{-1/2} \tag{S1.2}$$

This equation was used to describe FRAP data sets for all studied proteins: EGFP-Noggin1, EGFP-Noggin2, EGFP-Noggin4, the secreted version of EGFP-hep and EGFP-Wnt8. All data were renormalised to obtain array data of $C_z(t)$ values according to the equation (S1.2). Then a running average with the frame size of four points was calculated. Whole timeline for a group of observations was divided into 40 intervals. For every interval the average and the standard deviation were computed. Data sets were fitted to the equation:

$$y(x) = -a(b(x-c)-1)^{-0.5} \ ,$$

where b is a major parameter that determines the diffusion coefficient ($D = bw^2/2\pi$); a and c are parameters, which should neglect possible mistakes in normalization and calculation of dispersion at the very beginning of the FRAP curve. The following constraints were used:

$$0.9 \leq a \leq 1.1 \quad b \geq 0 \quad x_{s-1} < c < x_{s+1} \ ,$$

where s is a number of starting frames in the observation set (values 2, 3 or 4 were used). Limit-memory version of Broyden-Fletcher-Goldfarb-Shanno algorithm with Bounds (L-BFGS-B) was used for fitting. In particular we used BFGS functions included into scipy.optimize library.[7] The quality of fitting was estimated by the determination coefficient that was computed as followed:

$$R^2 = 1 - \frac{\sum_i (y_i^{(exp)} - y^{(fit)}(x_i))^2}{\sum_i (y_i^{(exp)} - \overline{y^{(exp)}})^2} \frac{n-1}{n-3}$$

As shown in Fig S10, FRAP of EGFP-Noggin4 protein could be fitted by the equation (S1.2) with the determination coefficient 0.79. However, this equation produces much worse fitting for the data obtained for other proteins, including EGFP-Noggin1, EGFP-Noggin2, the secreted version of EGFP-hep and EGFP-Wnt8. When individual samples of a specific set were fitted separately (data not shown), a fluctuation of the parameter b (that is proportional to D) was measured as a coefficient of variance (CV):

$$CV = \frac{\sigma(b)}{\bar{b}} \times 100\%$$

While CV was small enough for EGFP-Noggin4, this parameter strongly fluctuates in the cases of other proteins. Thus, if CV for EGFP-Noggin4 was about 20%, its values for the rest of proteins were much larger, reaching 115% for EGFP-Wnt8. This indicates that not only diffusion, but also other physical processes could contribute to the recovery of bleached proteins.

1.2. Model of FRAP process in the case of diffusion with adsorption on immobile binding centers.

To model diffusion of a protein in the condition of adsorption, the following kinetic scheme was studied:

$$\begin{aligned} A + B &\rightleftharpoons C \\ A_b + B &\rightleftharpoons C_b \end{aligned} \tag{S1.3}$$

where A is a rapidly diffusing protein, B is an immobile binding interactant, C is the complex of A bound to B; subscript "B" denotes a "bleached" protein (A can be bleached both in free and bound states). It is also assumed that both reactions have the same forward (k_1) and reverse (k_{-1}) reaction rate constants. Let D be the diffusion coefficient of A. B is immobile and let it be distributed uniformly with initial concentration σ. C is also immobile. Let concentrations of A, B and C be a, b and c, respectively. Since the forward reactions have the second order, the reverse reactions have the first order and $b = \sigma - c - c_b$, it follows that:

$$\begin{cases} \partial_t a = D\partial^2_{xx} a - k_1 a(\sigma - c - c_b) + k_{-1} c_b \\ \partial_t c = k_1 a(\sigma - c - c_b) - k_{-1} c \\ \partial_t a_b = D\partial^2_{xx} a_b - k_1 a_b(\sigma - c - c_b) + k_{-1} c_b \\ \partial_t c_b = k_1 a_b(\sigma - c - c_b) - k_{-1} c_b \end{cases} \tag{S1.4}$$

Let the protein A be distributed uniformly and be in equilibrium with the immobile molecules (B, C) before bleaching. As bleaching and diffusion do not influence on a global equilibrium, then we have:

1. sums $a + a_b$, $c + c_b$ are constants, only the ratio changes with bleaching/diffusion;
2. $\frac{([A]+[A_b])[B]}{[C]+[C_b]} = \frac{k_1}{k_{-1}} = K$ or in other notations $\frac{(a+a_b)(\sigma-c-c_b)}{c+c_b} = K$;
3. the concentration of free binding centers $\sigma - c - c_b$ is also constant.

By denoting $\sigma - c - c_b$ by the constant σ_{free}, we can reduce the system of equations (S1.4) and get:

$$\begin{cases} \partial_t a_b = D\partial^2_{xx} a_b - k_1 a_b \sigma_{free} + k_{-1} c_b \\ \partial_t c_b = k_1 a_b \sigma_{free} - k_{-1} c_b \end{cases} \tag{S1.5}$$

For this linear system, one can obtain an exact solution in Fourier form. If we replace $a_b = Ae^{\lambda t}e^{i\delta x}$, $c_b = Ce^{\lambda t}e^{i\delta x}$ in the system (S1.5) we obtain:

$$\begin{cases} A\lambda = -DA\delta^2 - k_1\sigma_{free}A + k_{-1}C \\ B\lambda = k_1\sigma_{free}A - k_{-1}C \end{cases} \tag{S1.6}$$

The eigenvalues $\lambda_{1,2}$ of the characteristic matrix have the form:

$$\lambda_{1,2} = -\frac{k_{-}1 + D\delta^2 + k_1\sigma_{free}}{2} \pm \frac{\sqrt{(k_{-1} + D\delta^2 + k_1\sigma_{free})^2 - 4D\delta^2 k_{-1}}}{2} \tag{S1.7}$$

Both eigenvalues are real and negative, therefore the homogenic stationary state is stable (the bleached region disappears). Assuming the concentration of the bleached protein at the reactor boundaries is constantly zero, we take the following Dirichlet boundary conditions:

$$a_b(0,t) = 0 \;\; a_b(l,t) = 0 \;\; c_b(0,t) = 0 \;\; c_b(l,t) = 0$$

Let an initial distribution of the bleached protein (both free and bound one) be of the form:

$$a_b(x,0) = a_0 \sin\frac{\pi x}{l}; \;\; c_b(x,0) = c_0 \sin\frac{\pi x}{l} \tag{S1.8}$$

Therefore, we get $\delta = \pi/l$. Finally, after finding eigenvectors for the system (S1.6), for a common solution of the system (S1.5) we obtain:

$$\begin{aligned} a_b(x,t) &= \sin\frac{\pi x}{l}\left[C_1 e^{\lambda_1 t} + C_2 e^{\lambda_2 t}\right] \\ c_b(x,t) &= \sin\frac{\pi x}{l}\left[C_1 \frac{k_1\sigma_{free}}{\lambda_1 + k_{-1}} e^{\lambda_1 t} + C_2 \frac{k_1\sigma_{free}}{\lambda_2 + k_{-1}} e^{\lambda_2 t}\right] \end{aligned} \tag{S1.9}$$

where C_1, C_2 are determined from the initial maximum concentrations a_0 and c_0 defined in the equation (S1.8). By definition, the fluorescent recovery in the middle-point of the reactor is a sum:

$$I(t) = a_b(l/2,t) + c_b(l/2,t) \tag{S1.10}$$

Combining the equations (S1.9) and (S1.10), we get:

$$\begin{gathered} I(t) = C_1\left(1+\frac{k_1\sigma_{free}}{\lambda_1+k_{-1}}\right)e^{\lambda_1 t} + C_2\left(1+\frac{k_1\sigma_{free}}{\lambda_2+k_{-1}}\right)e^{\lambda_2 t} \\ A_{1,2} = C_{1,2}\left(1+\frac{k_1\sigma_{free}}{\lambda_{1,2}+k_{-1}}\right) \end{gathered} \tag{S1.11}$$

$I(t)$ has a form of a linear combination of two exponents with characteristic times $-1/\lambda_1$, $-1/\lambda_2$ (slow and fast, respectively). For these times one can obtain asymptotic estimations. Let represent equation (S1.7) in the following form:

$$\lambda_{1,2} = -\frac{v}{2} \pm \frac{\sqrt{v^2-w}}{2}; \;\; v = k_{-1} + D\delta^2 + k_1\sigma_{free}; \;\; w = 4D\delta k_{-1} \tag{S1.12}$$

Under conditions of our experiment $D\delta^2 \approx 1$ and $k_{-1} << 1$, so $w < v$. Next, we expand equation (S1.12) to a Taylor series by w:

$$\lambda_{1,2} = [\lambda_{1,2}]_{w=0} + \left[\frac{\partial\lambda_{1,2}}{\partial w}\right]_{w=0} w + o(w^2)$$

Taking into account, that

$$[\lambda_{1,2}]_{w=0} = -v\left(\frac{1}{2}\mp\frac{1}{2}\right), \quad \left[\frac{\partial\lambda_{1,2}}{\partial w}\right]_{w=0} \mp \frac{1}{4v},$$

we get:

$$\lambda_1 \approx -\frac{w}{4v}, \quad \lambda_2 \approx -(v-\frac{w}{4v})$$

Using the assumption that $w << v$, we finally get for λ_2:

$$\lambda_2 \approx -v$$

In the initial notations defined by equations (S1.12) for two characteristic times we have:

$$\begin{aligned} \tau_1 &= -1/\lambda_1 \approx 1/k_{-1} + \frac{\sigma_{free}/K+1}{D}\frac{l^2}{\pi^2} \\ \tau_2 &= -1/\lambda_2 \approx \frac{1}{k_{-1}+D\pi^2/l^2+k_1\sigma_{free}} \end{aligned} \tag{S1.13}$$

Applying it to our system, we expect k_{-1} to be around 0.01 s^{-1}, K — around 10^{-7} M, l — around 10 μm, σ_{free} — around 10^{-8} M and D — around 10^{-8} cm^2/s. As the concentrations and constants are in these ranges, the characteristic times defined in the equations (S1.13) have exact physical meaning. The first characteristic time is defined mostly by complex lifetime ($1/k_{-1}$) and can be designated as "adsorption characteristic time". The second characteristic time is defined by spatial and diffusion characteristics (l^2/D) and can be designated as "diffusion characteristic time". At large t values the solution is reduced to a single exponent with the large characteristic time. Under the experimental conditions "adsorption characteristic time" is much larger than "diffusion characteristic time". As a result, long-term characteristic time of FRAP kinetics reflects a lifetime of the complex.

Note, that without loss of generality, asymptotic equations (S1.11) can be used when boundary conditions differ from a simple sinusoidal form (S1.8). We do not prove it rigorously, however we have tested the equations (S1.12),(S1.13) on the set of numerical solutions of the equations (S1.5). These tests show that the equations (S1.12),(S1.13) well describe numerical data under different parameters and under an initial Gaussian distribution of the bleached proteins.

The rigorous solution of the equations (S1.5) could be found in the papers of Tardy and McGrath[8,9] but working with such a form is uncomfortable. In the first study[8] the equations (S1.5) described the diffusion process of actin monomers accompanied with actin polymerisation and similar conclusions were drawn. Two regimes were isolated: "diffusion regime", when the fluorescence kinetics is determined by diffusion, and "turnover regime" when kinetics is determined by the equilibrium between unbound and cross-linked actin. In our case two regimes with different characteristic times also could be selected.

The common trends observed for completely different systems significantly reinforce the result. Also one may note that the model similar to (S1.5) was used for the determination of binding parameters (theoretically) in the Carrero's review.[10] The most self-consistent theory of reaction-with-adsorption systems was developed in McNally's group.[11] Analysing the equation (S1.5) authors theoretically isolated three possible simplified regimes: "pure-diffusion dominant", "effective diffusion" and "reaction dominant". Our model objects (adsorbed morphogens) resemble the last one because the FRAP characteristic time is determined mostly by the dissociation rate constant.

1.3. Effective diffusion coefficient

To introduce an effective diffusion coefficient correctly, one may focus on a single time dependency of C_z for EGFP-Noggin4 and the theoretical curve that is shown at Fig. 4b. The theoretical curve was plotted according to the equation (S1.2) using parameters $w = 14$, $D = 1.1\mu m^2/s$. Obviously, the term $4\pi D/w^2$ in the equation (S1.2) equals to the reverse time of $(1-1/e)$ recovery (Fig. 4b). The value $(1-1/e)$ equals approximately 2/3, so we denote this reverse time as $\tau_{2/3}$. When the curve could not be fitted by the equations (S1.1),(S1.2) correctly, then the effective diffusion coefficient could be introduced as:

$$D_E = (e^2-1)w^2/4\pi\tau_{2/3} \tag{S1.14}$$

Suggest $(e^2-1)/4\pi \approx 1/2$; then the simple form for the effective coefficient is:

$$D_E = w^2/2\tau_{2/3} \tag{S1.15}$$

The effective diffusion coefficient determined by the equations (S1.15) appears to be equal to the real diffusion coefficient when diffusion is Fickian. Thus, this equation could be applied to an arbitrary FRAP kinetics. Accordingly, we used the equation (S1.15) to interpret FRAP kinetics when a diffusion was accompanied by adsorption. Notably, a similar equation was used to assess "effective" or "apparent" diffusion coefficients of proteins that interact with a cell membrane.[10] An observation error for effective diffusion coefficient calculated by the equation (S1.15) can be calculated by this:

$$\Delta D = \frac{1}{4\pi}\sqrt{\left(\frac{2w}{\tau_{2/3}\Delta w}\right)^2 + \left(\frac{w^2}{\tau_{2/3}^2}\Delta\tau_{2/3}\right)^2}$$

For our experiments, we used the value of 2 μm as observation error of bleached region size (Δw) and as an observation error of recovery time ($\Delta\tau$).

To determine $\tau_{2/3}$ we should fit the recovery kinetics of adsorbed proteins with a correct equation. As we show in Supplementary Materials and Methods, 1.2, the recovery kinetics for proteins that are adsorbed at the cell surface should have a two-exponential form. The equation (S1.11) could be rewritten using parameters of a fit (a, c, τ_1, τ_2):

$$y(x) = -a \cdot \exp(-(x-c)/\tau_1) - (1-a)\cdot\exp(-(x-c)/\tau_2)$$

Thus, we fit our FRAP data with this double-exponential curves using same L-BFGS algorithm with constraints. We considered τ_1 to be the diffusion-driven recovery time and it could not be greater than 50 s, whereas τ_2 was considered to be the adsorption-driven recovery time and it could not be less than 50 s. All data fitted by this way are presented at Fig. 5. This kind of a fit is much better for all proteins than a fit presented at Fig. S10, because the values of the determination coefficients are around 0.9 for all experiments. Notably, the double-exponential equation fits Noggin4 experimental points slightly better than Ellenberg's equation. This indicates that Noggin4 nevertheless demonstrates a weak binding to some components of the extracellular matrix, without a significant effect on protein diffusivity. Theoretical curves plotted over FRAP data (Fig. 5) were used for determination of $\tau_{2/3}$, whereas the size of a bleached zone, w, was set by FRAP procedure. These $\tau_{2/3}$ and w values were used to compute D_E.

2. Mathematical analysis of FRAP data

3D structure of human Noggin homodimer in a complex with BMP2 was described earlier.[12] To determine the shapes of Noggin2 and Noggin4 homodimers, we used x-ray coordinates of human Noggin (PDB ID: 1M4U; doi:10.2210/pdb1M4U/pdb) as a reference structure and the method of homological modelling based on MODELLER software to construct the 3D models of these homodimers.[13]

To test the stability of homological models of Noggin proteins, all structures from this stage were simulated in the molecular dynamics. The coordinates of constructed homodimers were put into the boxes of size 10×× 14 nm and then the systems were solved with the sufficient amount of TIP3P water molecules and Na^+/Cl^- ion pairs were added to concentration of 150 mM. All-atom OPLS force field was used in topology.[14] MD calculations were performed in GROMACS package[15] with "stochastic

dynamic” integrator and Parrinello-Rahman barostate. The step of 2 fs was used in simulations as well as constraints for all valence bond lenghts. The structure equilibrated after 100 ns of simulation was used in further calculations. As a result, the shapes of Noggin2 and Noggin4 were determined by using built models (Fig. S11).

Free diffusion coefficients for all proteins were calculated by means of Hydropro software,[16] which considers only the shape of a molecule but not intramolecular mobility. This type of estimation is rather rough and, thus, we did not consider detailed structure of the linker between EGFP and the core protein. In our model EGFP protein was simply placed near the N-terminus of every Noggin monomers in the dimer (see Fig. S11 left).

As we were unable to find any information about viscosity within the intercellular space of *Xenopus laevis* embryos, we performed calculation of diffusion coefficients at different values of viscosity. The obtained dependency of the diffusion coefficient on viscosity is linear in double-logarithmic coordinates (see, as an example of these calculations, the diagram for EGFP-Noggin4 on Fig. S11b). By means of this diagram, we determined that the diffusion coefficient of EGFP-Noggin4, which was measured with FRAP technique in a living embryo (7.1 $\mu m^2/s$, see Table 1), corresponded to the diffusion coefficient of the same protein diffusing freely in liquid with the viscosity value of 0.05 Ps. This viscosity is very close to the viscosity of blood[3] and thus could be considered to be quite close to the viscosity within the intercellular space. In turn, this indicates that, by contrast to other Noggins, whose theoretic diffusivity exceeds their actual diffusivity in a living embryo more than in 40 times (see Table 1), the diffusion of Noggin4 in living embryo occurs almost freely, without significant interactions with HSPGs or other molecules in the intercellular space.

3. Estimation of the Noggin4-Wnt8 complex formation parameters by the *in vivo* imaging

Here we took advantage of the model described in Supplementary Materials and Methods, 1.2 to estimate the dissociation constant of the Noggin4-Wnt8 protein complex in a living embryo on the basis of FRAP data.

Let EGFP-Noggin4 be the rapidly diffusing protein A and let TagRFP-Wnt8 bound to HSPG be the immobile binding interactant **B** (S1.3). The idea of the developed approach is based on the fact that, as it is demonstrated above, the dissociation constant is determined by the ratio of amplitudes of the fast (diffusion, A_1) and the slow (adsorption, A_2) components of FRAP kinetics (see Fig. 4c). In particular, it results in different shapes of the theoretical curves built by means of the equations (S1.9) and describes FRAP kinetics of EGFP-Noggin4 in the presence of equal concentration of “immobile” TagRFP-Wnt8 at different dissociation constants of their protein complex (Fig. S13). In turn, having a set of experimental points of FRAP kinetics and fitting them to a curve built by means of the equations (S1.9), one may resolve an opposite task: to estimate the dissociation constant, which corresponds to this fitting curve.

The fluorescence intensity kinetics after photobleaching of the area where Noggin4 and Wnt8 are co-localised is shown at Fig. 9. This experimental data were fitted with the equations (S1.9). One can address the main system of equations (S1.5) and its initial conditions (S1.8) to observe all the parameters, which should be found. The concentration of Wnt8 and Noggin4 was measured from images and substituted into σ_{free}, a_0 in these equations. We take D of free Noggin4 (see Table 1) as the free diffusion coefficient D in these equations. Then, the rest parameters k_1, k_{-1} and their ratio (the dissociation constant) could be assessed by fitting. In Fig. S13 a series of curves which were plotted using our known parameters and different K values is shown. These experimental curves are similar to the red one in Fig. S13. Whereas the diffusion-driven kinetics (A_1) is much smaller than the adsorption-driven one (A_2), it is possible to make only an upper estimate of A_1. It means that the value of K could be of the order of 10^{-7} M or less. On Fig. S15 we show the fit of the averaged experimental curve with $K = 10^{-7}$ M. The fitting procedure was the same as described in Supplementary Materials and Methods, 1.1.

4. Estimation of Noggin4 influence on the Wnt signalling gradient formation.

4.1. Estimation of the endogenous Noggin4 concentration.

At first, we assessed the concentration of the endogenous Noggin4 protein in the intracellular space of living embryos. To this end, we estimated with qRT-PCR by how many times the concentration of *EGFP-Noggin4* mRNA in embryos that were analysed in our FRAP experiments was higher than the concentration of the endogenous *Noggin4* mRNA. To perform this analysis, we collected five replicated groups of the animal cap ectodermal explants (10 explants in each group) excised from different batches of the early gastrula wild-type embryos and from their siblings preliminary injected with EGFP-Noggin4 mRNA in the same way as it was done for FRAP experiments. The ectodermal explants were taken given the fact that, unlike the wild-type embryos, their microinjected siblings may contain Noggin4 templates not only in the ectodermal layer but throughout the embryo. The relative concentrations of *EGFP-Noggin4* and the endogenous *Noggin4* mRNA in these groups of explants were compared by qRT-PCR with the following pairs of primers to *EGFP* and *Noggin4* (these pairs of primers were preliminary selected as providing very close to identical efficiency of PCR from EGFP and Noggin4 templates):

EGFP Forward 5’-GGCGACGTAAACGGCCACA and
EGFP Reverse 5’-CTGCACGCCGTAGGTCAGG;

Nog4 Forward 5'-TGGTTGGTGCAGAGGGCCAG and
Nog4 Reverse 5'-CATGCCAGGTGGCCAGGAGC

As a result, we established that the concentration of the endogenous Noggin4 mRNA was on an average 20 times lower than the concentration of the synthetic EGFP-Noggin4 mRNA in embryos used for the FRAP experiments (Fig. S16).

Thus, assuming that the effectiveness of the endogenous Noggin4 mRNA translation could not be lower than that of the longer EGFP-Noggin4 mRNA, we concluded that the concentration of the endogenous Noggin4 protein could not be less than 1/20 of EGFP-Noggin4. Given that the concentration of the latter protein was estimated as 1 μM (see above), one may estimate the concentration of the endogenous Noggin4 protein in the intercellular space as $5{\cdot}10^{-8}$ M.

4.2. Studying by a static mathematical modeling of the ability of endogenous Noggin4 to influence Wnt8 signaling in the Xenopus laevis embryo.

For simplicity, we supposed the total concentration of Frizzled8 receptors that are able to bind Wnt8 to be close to that of Noggin4 — $5{\cdot}10^{-8}$ M — throughout the neurectoderm. In contrast, concentration of Wnt8 forms a posterior-anterior gradient; in its posterior maximum being also close to $5{\cdot}10^{-8}$ M but declining to the anterior end of the embryo according to the logistic sigmoid function, which fits the experimentally revealed the anterior-posterior gradient of Wnt/β-catenin signaling in the presumptive neural plate.

Finally, we took into account that Noggin4 and Frizzled8 bind to Wnt8 with the constants of 10^{-7} M (see above) and 10^{-8} M, respectively.

Given these initial conditions, we analysed if the change in Noggin4 concentration could exert influence on the established concentration gradient of Wnt8-Frizzled8 complex in the embryo, whose overall length was taken as 1000 microns. To this end, the following mathematical model was developed. Let two simultaneous competing reactions exists:

$$\begin{aligned} Wnt8 + Frizzled8 &\rightleftharpoons Wnt8 - Frizzled8 \\ Wnt8 + Noggin4 &\rightleftharpoons Wnt8 - Noggin4 \end{aligned} \tag{S4.1}$$

Let the concentrations of free Wnt8, Noggin4 and Frizzled8 be w, n and f, respectively. Let the full initial amount of every protein be known. Let denote the full amount by w_0, n_0 and f_0. So we get an equilibrium:

$$\frac{wn}{n_0 - n} = K_{wn} \quad \frac{wf}{f_0 - f} = K_{wf}$$

For the full amount of Wnt8, we obtain:

$$w + (n_0 - n) + (f_0 - f) = w_0$$

Combining last two equations we get:

$$\frac{(w_0 - n_0 - f_0 + f + n)n}{n_0 - n} = K_{wn} \quad \frac{(w_0 - n_0 - f_0 + f + n)f}{f_0 - f} = K_{wf} \tag{S4.2}$$

Finally, we have the system of two equations with two unknown variables: n and f. Thus, we can determine the amount of the Wnt8-Frizzled8 complex $(f_0 - f)$, which is proportional to the intensity of Wnt8 signalling.

As it is indicated above, we assumed that the concentration of Wnt8 declines from $5{\cdot}10^{-8}$ M at the posterior end of the embryo to zero at the anterior end according to the logistic sigmoid function:

$$w(x) = \frac{w_0}{1 + \exp(x/\Delta x)} \tag{S4.3}$$

where Δx is gradient steepness (Fig. 7). Then, given that the Frizzled8 and Noggin4 concentrations (f_0 and n_0 respectively) were postulated uniform along the body axis and equal to the posterior Wnt8 concentration of $5{\cdot}10^{-8}$ M, we calculated the concentration of the Wnt8-Frizzled8 $(f_0 - f)$ complex in each point along the axis according to the equations (S4.1)-(S4.3). (Fig. 7, black solid line). By means of the same equation, we investigated the influence on the Wnt8-Frizzled8 concentration gradient of different concentrations of Noggin4, thus modeling decrease or increase of the Noggin4 concentration in the experiments in injections of *Noggin4* MO and mRNA, respectively.

5. Calculation of K_d by the theoretical analysis of the Western Blotting data

The model used for analysis of WB data was based on an assumption that the following reactions take place:

1. Myc-Wnt8 binds to anti-Myc resin according to reaction W + R $\rightleftharpoons$ WR;
2. Flag-Wnt8 binds to Noggin4 according to reaction W + N $\rightleftharpoons$ WN;

Accordingly, four numerical parameters characterise these reactions:

1. active resin capacity $\rho = [R] + [WR]$;
2. active Noggin4 capacity $\eta = [N] + [WN]$;
3. K_d of WN complex, $K_1 = [W][N]/[WN]$;
4. K_d of WR complex, $K_2 = [W][R]/[WR]$.

When analysing data obtained with the control samples, one should consider only the reaction (i), whereas for the experimental samples both reactions, (i) and (ii), should be taken into account. Let designate [W], i.e. the total amount of Flag-Wnt8, as ω. Then, for the control samples, we can consider the concentration of the complex [WR] as:

$$[WR] = \frac{1}{2}\left(\rho + \omega + K_2 - \sqrt{(\rho + \omega + K_2)^2 - 4\rho\omega}\right) \tag{S5.1}$$

By means of this equation, the data obtained with the control samples allowed us to determine two of four parameters indicated above: ρ = 0.007 μM and K_2 = 0.1 μM.

By contrast to the process that takes place in the control samples, the reactions occurring in the experimental samples can be described with the following equation system, in which x is used to designate [WN] and y to designate [WR]:

$$\begin{cases} (\eta - x)(\omega - x - y) = K_1 x \\ (\rho - y)(\omega - x - y) = K_2 y \end{cases} \tag{S5.2}$$

Parameters that determine binding of Myc-Noggin4 to Flag-Wnt8 (i.e. η and K_1), can be calculated from the curve describing the dependency of the total amount of bound Flag-Wnt8 (i.e. $[WN] + [WR] = x + y$) from the amount of the unbound Flag-Wnt8 (i.e. $\omega - [WN] + [WR]$) **(S6A Fig)**. This curve was built by fitting the experimental points, which were obtained as a result of the processing of WB images by ImageJ software, to the appropriate solution of the system of equations (S5.1),(S5.2). Two of four parameters in this system (K_2 and ρ) were determined from the data of the control experiments, in which Flag-Wnt8 was bound with pure anti-Myc resin. Two other parameters (η and K_1) were picked up by fitting the experimental data to the solution of the equations (S5.1),(S5.2). During this, the system (S5.2) was converted into an implicit equation (below) and solved numerically.

$$\Phi(y) = (\eta - \omega + \frac{K_2 y}{\rho - y})K_2 y - K_1\left((\omega - y)(\rho - y) - K_2 y\right) \tag{S5.3}$$

By this way, we determined the value of K_1 (K_d of Noggin4-Wnt8) as approximately 10 nM, which is in a satisfactory agreement with the upper limit of the constant estimated in FRAP experiments.

Supplementary references

1. Eroshkin, F. M., Ermakova, G. V., Bayramov, A. V. & Zaraisky, A. G. Multiple noggins in vertebrate genome: cloning and expression of noggin2 and noggin4 in Xenopus laevis. *Gene expression patterns : GEP* **6**, 180–6 (2006).

2. Bayramov, A. V. *et al.* Novel functions of Noggin proteins: inhibition of Activin/Nodal and Wnt signaling. *Development (Cambridge, England)* **138**, 5345–56 (2011).

3. Få hræus, R. & Lindqvist, T. The Viscosity of the Blood in Narrow Capillary Tubes. *Am J Physiol – Legacy Content* **96**, 562–568 (1931).

4. Keith, A. D. & Snipes, W. Viscosity of Cellular Protoplasm. *Science* **183**, 5–7 (1974).

5. Ivanova, A. S., Tereshina, M. B., Ermakova, G. V., Belousov, V. V. & Zaraisky, A. G. Agr genes, missing in amniotes, are involved in the body appendages regeneration in frog tadpoles. *Sci Rep* **3**, 1279 (2013).

6. Ellenberg, J. *et al.* Nuclear membrane dynamics and reassembly in living cells: targeting of an inner nuclear membrane protein in interphase and mitosis. *The Journal of cell biology* **138**, 1193–206 (1997).

7. Morales, J. L. & Nocedal, J. Remark on "algorithm 778: L-BFGS-B: Fortran subroutines for large-scale bound constrained optimization". *ACM Transactions on Mathematical Software* **38**, 1–4 (2011).

8. Tardy, Y., McGrath, J. L., Hartwig, J. H. & Dewey, C. F. Interpreting photoactivated fluorescence microscopy measurements of steady-state actin dynamics. *Biophysical journal* **69**, 1674–82 (1995).

9. McGrath, J. L., Tardy, Y., Dewey, C. F., Meister, J. J. & Hartwig, J. H. Simultaneous measurements of actin filament turnover, filament fraction, and monomer diffusion in endothelial cells. *Biophysical journal* **75**, 2070–8 (1998).

10. Carrero, G., McDonald, D., Crawford, E., De Vries, G. & Hendzel, M. J. Using FRAP and mathematical modeling to determine the in vivo kinetics of nuclear proteins. *Methods* **29**, 14–28 (2003).

11. Sprague, B. L., Pego, R. L., Stavreva, D. a. & McNally, J. G. Analysis of binding reactions by fluorescence recovery after photobleaching. *Biophysical journal* **86**, 3473–95 (2004).

12. Groppe, J. *et al.* Structural basis of BMP signalling inhibition by the cystine knot protein Noggin. *Nature* **420**, 636–42 (2002).

13. Sali, A. & Blundell, T. L. Comparative protein modelling by satisfaction of spatial restraints. *Journal of molecular biology* **234**, 779–815 (1993).

14. Jorgensen, W. L., Maxwell, D. S. & Tirado-Rives, J. Development and testing of the OPLS all-atom force field on conformational energetics and properties of organic liquids. *Journal of the American Chemical Society* **118**, 11225–11236 (1996).

15. Hess, B., Kutzner, C., van der Spoel, D. & Lindahl, E. GROMACS 4: Algorithms for Highly Efficient, Load-Balanced, and Scalable Molecular Simulation. *Journal of Chemical Theory and Computation* **4**, 435–447 (2008).

16. Garcia De La Torre, J., Huertas, M. L. & Carrasco, B. Calculation of hydrodynamic properties of globular proteins from their atomic-level structure. *Biophysical Journal* **78**, 719–730 (2000).
